# Supplementary material for: QstR-dependent regulation of natural competence and type VI secretion in Vibrio cholerae
Source: Nucleic Acids Res. 2018 Aug 8;46(20):10619–34. doi: 10.1093/nar/gky717 (PMC6237807; doi:10.1093/nar/gky717)
Supplement: Supplementary Data [file gky717_supplemental_files.zip › Jaskolska_revision_Supplementary Material.pdf]

Supplementary Material

QstR-dependent regulation of natural competence and type VI secretion in *Vibrio cholerae*

Milena Jaskólska, Sandrine Stutzmann, Candice Stoudmann and Melanie Blokesch

Supplementary results

Transposon mutagenesis to identify the putative *comEA* regulator

In order to identify the missing link between QstR and *comEA* expression we performed transposon mutagenesis screens using the *mariner*-based transposon (1). Mutagenesis was performed in a strain that carried a *comEA-lacZ* transcriptional reporter fusion and an arabinose-inducible copy of *tfoX* (Tn*tfoX*). Two independent screens were performed in the presence of *tfoX* induction, and *comEA* expression was evaluated by monitoring LacZ production (i.e. blue color development of colonies on plates supplemented with X-gal). In the first attempt we screened ~ 20,000 colonies and identified all transposon insertions that resulted in decreased LacZ production. In the second screen we assessed ~ 60,000 colonies, but to try and exclude false-positives due to growth defective mutants, we excluded small white colonies. Altogether, we identified multiple insertions in genes encoding known *comEA* regulators such as *qstR, hapR, cytR* and *cyaA* that abolished LacZ production, as well as numerous insertions in the *comEA-lacZ* reporter and Tn*tfoX* (Table S4). Notably, we also identified a small set of hits in genes not previously linked to competence regulation (Table S4).

None of the genes identified in the screens appeared to be expressed in a *qstR*-dependent manner, as evidenced by the RNA-seq data (Supplementary file 1 and 2). Additionally, some of the genes identified probably act indirectly, such as *varA* (Table S4), which encodes a response regulator required for HapR production (2). Since HapR is required for *comEA* expression we did not investigate this mutant further. Nevertheless, we attempted to investigate whether any of the other genes might still encode a factor involved in either *comEA* or competence regulation. Therefore, for the genes that were

identified more than once in the screens, we made clean deletions in the Tn*tfoX comEA-lacZ* reporter strain and confirmed that, in all cases except *VC0767*, they resulted in decreased *comEA-lacZ* expression (Fig. S14A). Next, we deleted these validated genes in the A1552-Tn*tfoX* strain and tested them in the chitin-independent transformation assay (Fig. S14B and C), as discussed below.

In the set of genes identified from the first screen, a transposon insertion in *VC0539* was identified only once but since the same screen also found an insertion in *VC0541*, within the same operon and this operon appears to be induced by TfoX (Supplementary file 1), the mutant was included in the analysis. However, transformation of a Δ*VC0539* strain was not obviously impaired. In contrast, deletion of the other genes identified in this screen i.e. *gidA* (*mnmG*), *trmE* (*mnmE*) and *miaA* all resulted in significantly reduced transformation frequencies (Fig. S14B). These genes encode homologs of enzymes involved in tRNA modification, are functionally related (3-5) and their inactivation leads to an increased frequency of translational frameshifts in *E. coli* (6). In *V. cholerae* deletion of these genes also results in slower growth and all three were previously identified in a transposon screen attempting to find novel regulators of *tcpA* expression (7). Thus we conclude that none of these genes encodes a specific regulator or either *comEA* or competence.

Finally, deletion of *flgD*, which encodes a flagellar rod protein (8) also significantly affected transformation frequency (~ 100 fold, Fig. S14C). However, this effect was independent of the defect in motility, since the transformation of two non-motile strains carrying deletions of either *flaA* (flagellin (9)) or *pomB* (encoding the motor protein (10)) was unaffected (Fig. S14C, shaded box). Previously, it was shown that in the *flgD* mutant the expression of *hapR* is repressed (8). We confirmed that HapR levels, and

consequently QstR levels, are lower in this strain by Western blotting (Fig. S14D). In support of the idea that reduced levels of QstR are responsible for the transformation defect of the *flgD* mutant, production of additional QstR (via Tn*qstR*) was sufficient to restore transformation to WT levels (Fig. S14E). Therefore, we conclude that the *flgD* deletion affects *comEA* expression by reducing HapR levels and, as a consequence, leads to lower production of QstR. A related transposon hit in *VC2067*, which encodes a MinD-like protein flagellar biosynthesis protein, also behaved in a similar manner, though its effect on transformation was less severe (Fig. S14A, C and E).

Table S1. Bacterial strains and plasmids used in this study.

Strain name	Genotype	Strain #	Source
Vibrio cholerae			
A1552	A1552 wild type, O1 El Tor Inaba; Rif^R	1	(11)
A1552-Tn*tfoX*	A1552 carrying mini-Tn7-*araC*-P_{BAD}-*tfoX* (Tn*tfoX*); Rif^R, $Gent^R$	1626	(12)
A1552-LacZ-Kan	A1552-*lacZ*::Kan; Rif^R,Kan^R	135	(13)
Δ*qstR*	A1552Δ*qstR* (*VC0396*); Rif^R	600	(14)
Δ*qstR*-Tn*tfoX*	A1552ΔqstR carrying mini-Tn7-*araC*-P_{BAD}-*tfoX* (Tn*tfoX*); Rif^R, $Gent^R$	1631	(14)
FRT-*qstR*-Tn*tfoX* [WT_qstR (FRT control)-Tn*tfoX*]	A1552::FRT::*qstR* carrying mini-Tn7-*araC*-P_{BAD}-*tfoX* (Tn*tfoX*); Rif^R, $Gent^R$	2490	(15)
FRT-*qstR*[P129T]-Tn*tfoX*	A1552-FRT::*qstR*[P129T] carrying mini-Tn7-*araC*-P_{BAD}-*tfoX* (Tn*tfoX*); Rif^R, $Gent^R$	2785	This study
FRT-*qstR*[P129A]-Tn*tfoX*	A1552-FRT::*qstR*[P129A] carrying mini-Tn7-*araC*-P_{BAD}-*tfoX* (Tn*tfoX*); Rif^R, $Gent^R$	6304	This study
FRT-*qstR*[R130A]-Tn*tfoX*	A1552-FRT::*qstR*[R130A] carrying mini-Tn7-*araC*-P_{BAD}-*tfoX* (Tn*tfoX*); Rif^R, $Gent^R$	2786	This study
FRT-*qstR*[L137A]-Tn*tfoX*	A1552-FRT::*qstR*[L137A] carrying mini-Tn7-*araC*-P_{BAD}-*tfoX* (Tn*tfoX*); Rif^R, $Gent^R$	6305	This study
FRT-*qstR*[R141A]-Tn*tfoX*	A1552-FRT::*qstR*[R141A] carrying mini-Tn7-*araC*-P_{BAD}-*tfoX* (Tn*tfoX*); Rif^R, $Gent^R$	6306	This study
FRT-*qstR*[D58A]-Tn*tfoX*	A1552-FRT::*qstR*[D58A] carrying mini-Tn7-*araC*-P_{BAD}-*tfoX* (Tn*tfoX*); Rif^R, $Gent^R$	2787	This study
FRT-*qstR*[D58E]-Tn*tfoX*	A1552-FRT::*qstR*[D58E] carrying mini-Tn7-*araC*-P_{BAD}-*tfoX* (Tn*tfoX*); Rif^R, $Gent^R$	2788	This study
Δ*qstR*-Tn*tfoX/*pBAD-*qstR*-N-strep	A1552Δ*qstR*-Tn*tfoX* carrying plasmid with *qstR* gene preceded by sequence encoding Strep-tagII® cloned into pBAD/Myc-HisA, arabinose inducible; Rif^R, $Gent^R$,Amp^R	2729	This study
Δ*qstR*-Tn*tfoX/*pBAD-*qstR*[P129T]-N-strep	A1552Δ*qstR*-Tn*tfoX* carrying plasmid with *qstR*[P129T] preceded by sequence encoding Strep-tagII® in pBAD/Myc-HisA vector, arabinose inducible; Rif^R, $Gent^R$,Amp^R	6330	This study
Δ*qstR*-Tn*tfoX* pBAD-*qstR*[P129A]-N-strep	A1552Δ*qstR*-Tn*tfoX* carrying plasmid with *qstR*[P129A] preceded by sequence encoding Strep-tagII® in pBAD/Myc-HisA vector, arabinose inducible; Rif^R, $Gent^R$,Amp^R	6331	This study
Δ*qstR*-Tn*tfoX/*pBAD-*qstR*[R130A]-N-strep	A1552Δ*qstR*-Tn*tfoX* carrying plasmid with *qstR*[R130A] preceded by sequence encoding Strep-tagII® in pBAD/Myc-HisA vector, arabinose inducible; Rif^R, $Gent^R$,Amp^R	6332	This study

Δ*qstR*-Tn*tfoX/*pBAD-*qstR*[L137A]-N-strep	A1552Δ*qstR*-Tn*tfoX* carrying plasmid with *qstR*[L137A] preceded by sequence encoding Strep-tagII® in pBAD/Myc-HisA vector, arabinose inducible; Rif^R, $Gent^R$,Amp^R	6333	This study
Δ*qstR*-Tn*tfoX/*pBAD-*qstR*[R141A]-N-strep	A1552Δ*qstR*-Tn*tfoX* carrying plasmid with *qstR*[R141A] preceded by sequence encoding Strep-tagII® in pBAD/Myc-HisA vector, arabinose inducible; Rif^R, $Gent^R$,Amp^R	6334	This study
A1552-Tn*vdcA*	A1552 carrying mini-Tn7-*araC*-P_{BAD}-*vdcA* (Tn*vdcA*); Rif^R, $Gent^R$	2947	(16)
A1552-Tn*cdpA*	A1552 carrying mini-Tn7-*araC*-P_{BAD}-*cdpA* (Tn*cdpA*); Rif^R, $Gent^R$	2948	(16)
FRT-*qstR*	A1552-FRT::*qstR*	6307	This study
FRT-*qstR*-Tn*vdcA*	A1552-FRT::*qstR* carrying mini-Tn7-*araC*-P_{BAD}-*vdcA* (Tn*vdcA*); Rif^R, $Gent^R$	6308	This study
FRT-*qstR*-Tn*cdpA*	A1552-FRT::*qstR* carrying mini-Tn7-*araC*-P_{BAD}-*cdpA* (Tn*cdpA*); Rif^R, $Gent^R$	6309	This study
FRT-*qstR*[P129T]	A1552-FRT::*qstR*[P129T]	6310	This study
FRT-*qstR*[P129T]-Tn*vdcA*	A1552-FRT::*qstR*[P129T] carrying mini-Tn7-*araC*-P_{BAD}-*vdcA* (Tn*vdcA*); Rif^R, $Gent^R$	6311	This study
FRT-*qstR*[P129T]-Tn*cdpA*	A1552-FRT::*qstR*[P129T] carrying mini-Tn7-*araC*-P_{BAD}-*cdpA* (Tn*cdpA*); Rif^R, $Gent^R$	6312	This study
i*tfoX*	A1552Δ*lacZ*::i*tfoX* (*araC*-P_{BAD}-*tfoX)*; Rif^R	5594	This study
i*tfoX*Δ*qstR*	A1552Δ*lacZ*::i*tfoX* Δ*qstR*; Rif^R	5831	This study
i*tfoX*-Tn*vdcA*	A1552Δ*lacZ*::i*tfoX* carrying mini-Tn7-*araC*-P_{BAD}-*vdcA* (Tn*vdcA*); Rif^R, $Gent^R$	5832	This study
i*tfoX*-Tn*cdpA*	A1552Δ*lacZ*::i*tfoX* carrying mini-Tn7-*araC*-P_{BAD}-*cdpA* (Tn*cdpA*); Rif^R, $Gent^R$	5834	This study
i*tfoX*Δ*qstR*-Tn*vdcA*	A1552Δ*lacZ*::i*tfoX* Δ*qstR* carrying mini-Tn7-*araC*-P_{BAD}-*vdcA* (Tn*vdcA*); Rif^R, $Gent^R$	5833	This study
i*tfoX*Δ*qstR*-Tn*cdpA*	A1552Δ*lacZ*::i*tfoX* Δ*qstR* carrying mini-Tn7-*araC*-P_{BAD}-*cdpA* (Tn*cdpA*); Rif^R, $Gent^R$	5835	This study
i*tfoX*-FRT-*qstR*	A1552Δ*lacZ*::i*tfoX* FRT::*qstR*	5836	This study
i*tfoX*-FRT-*qstR*-Tn*vdcA*	A1552Δ*lacZ*::i*tfoX* FRT::*qstR* carrying mini-Tn7-*araC*-P_{BAD}-*vdcA* (Tn*vdcA*); Rif^R, $Gent^R$	5837	This study
i*tfoX*-FRT-*qstR*-Tn*cdpA*	A1552Δ*lacZ*::i*tfoX* FRT::*qstR* carrying mini-Tn7-*araC*-P_{BAD}-*cdpA* (Tn*cdpA*); Rif^R, $Gent^R$	5838	This study
i*tfoX*-FRT-*qstR*[P129T]	A1552Δ*lacZ*::i*tfoX* FRT::*qstR*[P129T]	5839	This study
i*tfoX*-FRT-*qstR*[P129T]-Tn*vdcA*	A1552Δ*lacZ*::i*tfoX* FRT::*qstR*[P129T] carrying mini-Tn7-*araC*-P_{BAD}-*vdcA* (Tn*vdcA*); Rif^R, $Gent^R$	5840	This study
i*tfoX*-FRT-*qstR*[P129T]-Tn*cdpA*	A1552Δ*lacZ*::i*tfoX* FRT::*qstR*[P129T] carrying mini-Tn7-*araC*-P_{BAD}-*cdpA* (Tn*cdpA*); Rif^R, $Gent^R$	5841	This study
A1552-Tn*tfoX*-strep	A1552 carrying mini-Tn7-*araC*-P_{BAD}-*tfoX* –strep (Tn*tfoX*-strep); Rif^R, $Gent^R$	3420	(16)
A1552-Tn	A1552 carrying mini-Tn7-empty; Rif^R, $Gent^R$	1998	This study
A1552-Tn*qstR*	A1552 carrying mini-Tn7-*araC*-P_{BAD}-*qstR* (Tn*qstR*); Rif^R, $Gent^R$	5501	This study

Δ*vpsA*Δ*hapR*-Tn	A1552Δ*vpsA*::FRT Δ*hapR*::FRT carrying mini-Tn7-empty; Rif^R, $Gent^R$	5489	This study
Δ*vpsA*Δ*hapR*-Tn*qstR*	A1552Δ*vpsA*::FRT Δ*hapR*::FRT carrying mini-Tn7-*araC*-P_{BAD}-*qstR* (Tn*qstR*); Rif^R, $Gent^R$	5631	This study
Δ*comM*	A1552Δ*comM* (*VC0032*); Rif^R	381	This study
Δ*comF*	A1552Δ*comF* (*VC2719*); Rif^R	555	(17)
Δ*comEC*	A1552Δ*comEC* (*VC1879*); Rif^R	519	(18)
ΔcomEA	A1552Δ*comEA* (*VC1917*); Rif^R	52	(19)
Δ*ligA2*	A1552Δ*ligA2* (*VC1542*); Rif^R	842	This study
Δ*VC1479*	A1552Δ*VC1479*::FRT; Rif^R	6313	This study
Δ*VC0033*	A1552Δ*VC0033*::FRT; Rif^R	6127	This study
Δ*VC0542*	A1552Δ*VC0542*; Rif^R	504	This study
Δ*vipA*	A1552Δ*vipA*::FRT (*VCA0107*); Rif^R	3042	(20)
Δ*comM*-Tn*tfoX*	A1552Δ*comM* carrying mini-Tn7-*araC*-P_{BAD}-*tfoX* (Tn*tfoX*); Rif^R, $Gent^R$	3274	This study
Δ*comF*-Tn*tfoX*	A1552 Δ*comF* carrying mini-Tn7-*araC*-P_{BAD}-*tfoX* (Tn*tfoX*); Rif^R, $Gent^R$	3142	(17)
Δ*comEC*-Tn*tfoX*	A1552Δ*comEC* carrying mini-Tn7-*araC*-P_{BAD}-*tfoX* (Tn*tfoX*); Rif^R, $Gent^R$	3134	(17)
Δ*comEA*-Tn*tfoX*	A1552Δ*comEA* carrying mini-Tn7-*araC*-P_{BAD}-*tfoX* (Tn*tfoX*); Rif^R, $Gent^R$	1645	(14)
Δ*ligA2*-Tn*tfoX*	A1552Δ*ligA2* carrying mini-Tn7-*araC*-P_{BAD}-*tfoX* (Tn*tfoX*); Rif^R, $Gent^R$	4997	This study
Δ*VC1479*-Tn*tfoX*	A1552Δ*VC1479*::FRT carrying mini-Tn7-*araC*-P_{BAD}-*tfoX* (Tn*tfoX*); Rif^R, $Gent^R$	6314	This study
Δ*VC0033*-Tn*tfoX*	A1552Δ*VC0033*::FRT carrying mini-Tn7-*araC*-P_{BAD}-*tfoX* (Tn*tfoX*); Rif^R, $Gent^R$	6129	This study
Δ*VC0542*-Tn*tfoX*	A1552Δ*VC0542*::FRT carrying mini-Tn7-*araC*-P_{BAD}-*tfoX* (Tn*tfoX*); Rif^R, $Gent^R$	6315	This study
Δ*vipA*-Tn*tfoX*	A1552Δ*vipA*::FRT carrying mini-Tn7-*araC*-P_{BAD}-*tfoX* (Tn*tfoX*); Rif^R, $Gent^R$	3000	(20)
Δ*qstR*-Tn*qstR*	A1552 Δ*qstR* carrying mini-Tn7-*araC*-P_{BAD}-*qstR* (Tn*qstR*); Rif^R, $Gent^R$	5503	This study
Δ*qstR*-Tn*qstR*[L137A]	A1552Δ*qstR* carrying mini-Tn7-*araC*-P_{BAD}-*qstR*[L137A] (Tn*qstR*[L137A]); Rif^R, $Gent^R$	5548	This study
i*tfoX*Δ*qstR*-Tn*qstR*	A1552Δ*lacZ*::i*tfoX* Δ*qstR* carrying mini-Tn7-*araC*-P_{BAD}-*qstR* (Tn*qstR*); Rif^R, $Gent^R$	6036	This study
i*tfoX*Δ*qstR*-Tn*qstR*[L137A]	A1552Δ*lacZ*::i*tfoX* Δ*qstR* carrying mini-Tn7-*araC*-P_{BAD}-*qstR*[L137A] (Tn*qstR*[L137A]); Rif^R, $Gent^R$	6037	This study
Δ*tfoX*-Tn*qstR*	A1552Δ*tfoX* carrying mini-Tn7-*araC*-P_{BAD}-*qstR* (Tn*qstR*); Rif^R, $Gent^R$	5575	This study
Δ*hapR*-Tn*qstR*	A1552Δ*hapR* carrying mini-Tn7-*araC*-P_{BAD}-*qstR* (Tn*qstR*); Rif^R, $Gent^R$	5502	This study
Δ*vasH*-Tn*qstR*	A1552Δ*vasH*::FRT carrying mini-Tn7-*araC*-P_{BAD}-*qstR* (Tn*qstR*); Rif^R, $Gent^R$	5504	This study
i*tfoX*Δ*qstR*Δ*hapR*-Tn*qstR*	A1552Δ*lacZ*::i*tfoX* Δ*qstR* Δ*hapR* carrying mini-Tn7-*araC*-P_{BAD}-*qstR* (Tn*qstR*); Rif^R, $Gent^R$	6419	This study

i*tfoX*Δ*qstR*Δ*dns*-Tn*qstR*	A1552Δ*lacZ*::i*tfoX* Δ*qstR* Δ*dns* carrying mini-Tn7-*araC*-P_{BAD}-*qstR* (Tn*qstR*); Rif^R, $Gent^R$	6420	This study
i*tfoX*Δ*qstR*Δ*hapR*Δdns-Tn*qstR*	A1552Δ*lacZ*::i*tfoX* Δ*qstR* Δ*hapR* Δ*dns* carrying mini-Tn7-*araC*-P_{BAD}-*qstR* (Tn*qstR*); Rif^R, $Gent^R$	6421	This study
Δ*VC0008*-Tn*tfoX*	A1552Δ*VC0008::FRT-cat-FRT* carrying mini-Tn7-*araC*-P_{BAD}-*tfoX* (Tn*tfoX*)*;* Rif^R, $Gent^R$	4887	This study
ΔVC*0216*-Tn*tfoX*	A1552Δ*VC0216::FRT-cat-FRT* carrying mini-Tn7-*araC*-P_{BAD}-*tfoX* (Tn*tfoX*)*;* Rif^R, $Gent^R$	4888	This study
ΔVC*0216peak*-Tn*tfoX*	A1552Δ*VC0216peak::FRT-cat-FRT* (deletion of the ChIP-seq peak) carrying mini-Tn7-*araC*-P_{BAD}-*tfoX* (Tn*tfoX*)*;* Rif^R, $Gent^R$	4889	This study
Δ*VC0264*-Tn*tfoX*	A1552Δ*VC0264::FRT-cat-FRT* carrying mini-Tn7-*araC*-P_{BAD}-*tfoX* (Tn*tfoX*)*;* Rif^R, $Gent^R$	4890	This study
Δ*dksA*-Tn*tfoX*	A1552Δ*dksA::FRT-cat-FRT* (*VC0596*) carrying mini-Tn7-*araC*-P_{BAD}-*tfoX* (Tn*tfoX*)*;* Rif^R, $Gent^R$	4891	This study
ΔVC*0813*-Tn*tfoX*	A1552Δ*VC0813::FRT-cat-FRT* carrying mini-Tn7-*araC*-P_{BAD}-*tfoX* (Tn*tfoX*)*;* Rif^R, $Gent^R$	4892	This study
ΔVC*0903*-Tn*tfoX*	A1552Δ*VC0903::FRT-cat-FRT* carrying mini-Tn7-*araC*-P_{BAD}-*tfoX* (Tn*tfoX*)*;* Rif^R, $Gent^R$	4893	This study
Δ*asnB*-Tn*tfoX*	A1552Δ*asnB::FRT-cat-FRT* (*VC0991*) carrying mini-Tn7-*araC*-P_{BAD}-*tfoX* (Tn*tfoX*)*;* Rif^R, $Gent^R$	4894	This study
ΔVC*1120*-Tn*tfoX*	A1552Δ*VC1120::FRT-cat-FRT* carrying mini-Tn7-*araC*-P_{BAD}-*tfoX* (Tn*tfoX*)*;* Rif^R, $Gent^R$	4895	This study
ΔVC*1203*-Tn*tfoX*	A1552Δ*VC1203::FRT-cat-FRT* carrying mini-Tn7-*araC*-P_{BAD}-*tfoX* (Tn*tfoX*)*;* Rif^R, $Gent^R$	4896	This study
ΔVC*1370*-Tn*tfoX*	A1552Δ*VC1370::FRT-cat-FRT* carrying mini-Tn7-*araC*-P_{BAD}-*tfoX* (Tn*tfoX*)*;* Rif^R, $Gent^R$	4897	This study
ΔVC*1423*-Tn*tfoX*	A1552Δ*VC1423::FRT-cat-FRT* carrying mini-Tn7-*araC*-P_{BAD}-*tfoX* (Tn*tfoX*)*;* Rif^R, $Gent^R$	4898	This study
ΔVC*1430*-Tn*tfoX*	A1552Δ*VC1430::FRT-cat-FRT* carrying mini-Tn7-*araC*-P_{BAD}-*tfoX* (Tn*tfoX*)*;* Rif^R, $Gent^R$	4899	This study
ΔVC*1527*-Tn*tfoX*	A1552Δ*VC1527::FRT-cat-FRT* carrying mini-Tn7-*araC*-P_{BAD}-*tfoX* (Tn*tfoX*)*;* Rif^R, $Gent^R$	4900	This study
ΔVC*1651*-Tn*tfoX*	A1552Δ*VC1651::FRT-cat-FRT* carrying mini-Tn7-*araC*-P_{BAD}-*tfoX* (Tn*tfoX*)*;* Rif^R, $Gent^R$	4901	This study
ΔVC*1729-30*-Tn*tfoX*	A1552Δ*VC1729-30::FRT-cat-FRT* carrying mini-Tn7-*araC*-P_{BAD}-*tfoX* (Tn*tfoX*)*;* Rif^R, $Gent^R$	4902	This study
ΔVC*1764*-Tn*tfoX*	A1552Δ*VC1764::FRT-cat-FRT* carrying mini-Tn7-*araC*-P_{BAD}-*tfoX* (Tn*tfoX*)*;* Rif^R, $Gent^R$	4903	This study
Δ*ruvB*-Tn*tfoX*	A1552Δ*ruvB::FRT-cat-FRT* (*VC1845*) carrying mini-Tn7-*araC*-P_{BAD}-*tfoX* (Tn*tfoX*)*;* Rif^R, $Gent^R$	4904	This study
ΔVC*2227*-Tn*tfoX*	A1552Δ*VC2227::FRT-cat-FRT* carrying mini-Tn7-*araC*-P_{BAD}-*tfoX* (Tn*tfoX*)*;* Rif^R, $Gent^R$	4905	This study
ΔVC*2320*-Tn*tfoX*	A1552Δ*VC2320::FRT-cat-FRT* carrying mini-Tn7-*araC*-P_{BAD}-*tfoX* (Tn*tfoX*)*;* Rif^R, $Gent^R$	4906	This study
ΔVC*2497*-Tn*tfoX*	A1552Δ*VC2497::FRT-cat-FRT* carrying mini-Tn7-*araC*-P_{BAD}-*tfoX* (Tn*tfoX*)*;* Rif^R, $Gent^R$	4907	This study
ΔVC*2757*-Tn*tfoX*	A1552Δ*VC2757::FRT-cat-FRT* carrying mini-Tn7-*araC*-P_{BAD}-*tfoX* (Tn*tfoX*)*;* Rif^R, $Gent^R$	4908	This study

ΔV*CA0536*-Tn*tfoX*	A1552Δ*VCA0536::FRT-cat-FRT* carrying mini-Tn7-*araC*-P_{BAD}-*tfoX* (Tn*tfoX*); Rif^R, $Gent^R$	4909	This study
ΔV*CA0571*-Tn*tfoX*	A1552Δ*VCA0571::FRT-cat-FRT* carrying mini-Tn7-*araC*-P_{BAD}-*tfoX* (Tn*tfoX*); Rif^R, $Gent^R$	4910	This study
ΔV*CA0691*-Tn*tfoX*	A1552Δ*VCA0691::FRT-cat-FRT* carrying mini-Tn7-*araC*-P_{BAD}-*tfoX* (Tn*tfoX*); Rif^R, $Gent^R$	4911	This study
ΔV*CA0773*-Tn*tfoX*	A1552Δ*VCA0773::FRT-cat-FRT* carrying mini-Tn7-*araC*-P_{BAD}-*tfoX* (Tn*tfoX*); Rif^R, $Gent^R$	4912	This study
ΔV*CA0781*-Tn*tfoX*	A1552Δ*VCA0781::FRT-cat-FRT* carrying mini-Tn7-*araC*-P_{BAD}-*tfoX* (Tn*tfoX*); Rif^R, $Gent^R$	4913	This study
Δ*vesA*-Tn*tfoX*	A1552Δ*vesA::FRT-cat-FRT* (*VCA0803*) carrying mini-Tn7-*araC*-P_{BAD}-*tfoX* (Tn*tfoX*); Rif^R, $Gent^R$	4914	This study
ΔV*CA1012*-Tn*tfoX*	A1552Δ*VCA1012::FRT-cat-FRT* carrying mini-Tn7-*araC*-P_{BAD}-*tfoX* (Tn*tfoX*); Rif^R, $Gent^R$	4915	This study
ΔV*CA1048*-Tn*tfoX*	A1552Δ*VCA1048::FRT-cat-FRT* carrying mini-Tn7-*araC*-P_{BAD}-*tfoX* (Tn*tfoX*); Rif^R, $Gent^R$	4916	This study
Δ*trmY*-Tn*tfoX*	A1552Δ*trmY::FRT-cat-FRT* (*VCA1059*) carrying mini-Tn7-*araC*-P_{BAD}-*tfoX* (Tn*tfoX*); Rif^R, $Gent^R$	4917	This study
comEA-lacZ Tn*tfoX*	A1552Δ*lacZ* carrying transcriptional *comEA-lacZ* fusion and mini-Tn7-*araC*-P_{BAD}-*tfoX* (Tn*tfoX*); Rif^R, $Gent^R$	6088	This study
*comEA-lacZ*Δ*qstR*-Tn*tfoX*	A1552Δ*lacZ* Δ*qstR::FRT-aph-FRT* carrying transcriptional *comEA-lacZ* fusion and mini-Tn7-*araC*-P_{BAD}-*tfoX* (Tn*tfoX*); Rif^R, $Gent^R$, Kan^R	6335	This study
*comEA-lacZ*Δ*VC0008*-Tn*tfoX*	A1552Δ*lacZ*Δ*VC0008::FRT-cat-FRT* carrying transcriptional *comEA-lacZ* fusion and mini-Tn7-*araC*-P_{BAD}-*tfoX* (Tn*tfoX*); Rif^R, $Gent^R$, Cm^R	6336	This study
comEA-lacZ ΔVC0216-Tn*tfoX*	A1552Δ*lacZ*Δ*VC0216::FRT-cat-FRT* carrying transcriptional *comEA-lacZ* fusion and mini-Tn7-*araC*-P_{BAD}-*tfoX* (Tn*tfoX*); Rif^R, $Gent^R$, Cm^R	6337	This study
*comEA-lacZ*ΔV*C0216peak*-Tn*tfoX*	A1552Δ*lacZ*Δ*VC0216peak::FRT-cat-FRT* (deletion of the ChIP-seq peak) carrying transcriptional *comEA-lacZ* fusion and mini-Tn7-*araC*-P_{BAD}-*tfoX* (Tn*tfoX*); Rif^R, $Gent^R$, Cm^R	6338	This study
*comEA-lacZ*Δ*VC0264*-Tn*tfoX*	A1552Δ*lacZ*Δ*VC0264::FRT-cat-FRT* carrying transcriptional *comEA-lacZ* fusion and mini-Tn7-*araC*-P_{BAD}-*tfoX* (Tn*tfoX*); Rif^R, $Gent^R$, Cm^R	6339	This study
*comEA-lacZ*Δ*dksA*-Tn*tfoX*	A1552Δ*lacZ*Δ*dksA::FRT-cat-FRT* (*VC0596*) carrying transcriptional *comEA-lacZ* fusion and mini-Tn7-*araC*-P_{BAD}-*tfoX* (Tn*tfoX*); Rif^R, $Gent^R$, Cm^R	6340	This study
*comEA-lacZ*ΔV*C0813*-Tn*tfoX*	A1552Δ*lacZ*Δ*VC0813::FRT-cat-FRT* carrying transcriptional *comEA-lacZ* fusion and mini-Tn7-*araC*-P_{BAD}-*tfoX* (Tn*tfoX*); Rif^R, $Gent^R$, Cm^R	6351	This study
*comEA-lacZ*ΔV*C0903*-Tn*tfoX*	A1552Δ*lacZ*Δ*VC0903::FRT-cat-FRT* carrying transcriptional *comEA-lacZ* fusion and mini-Tn7-*araC*-P_{BAD}-*tfoX* (Tn*tfoX*); Rif^R, $Gent^R$, Cm^R	6350	This study

*comEA-lacZ*Δ*asnB*-Tn*tfoX*	A1552Δ*lacZ*Δ*asnB::FRT-cat-FRT* (*VC0991*) carrying transcriptional *comEA-lacZ* fusion and mini-Tn7-*araC*-P_{BAD}-*tfoX* (Tn*tfoX*); Rif^R, $Gent^R$, Cm^R	6349	This study
*comEA-lacZ*ΔVC*1120*-Tn*tfoX*	A1552Δ*lacZ*Δ*VC1120::FRT-cat-FRT* carrying transcriptional *comEA-lacZ* fusion and mini-Tn7-*araC*-P_{BAD}-*tfoX* (Tn*tfoX*); Rif^R, $Gent^R$, Cm^R	6348	This study
*comEA-lacZ*ΔVC*1203*-Tn*tfoX*	A1552Δ*lacZ*Δ*VC1203::FRT-cat-FRT* carrying transcriptional *comEA-lacZ* fusion and mini-Tn7-*araC*-P_{BAD}-*tfoX* (Tn*tfoX*); Rif^R, $Gent^R$, Cm^R	6347	This study
*comEA-lacZ*ΔVC*1370*-Tn*tfoX*	A1552Δ*lacZ*Δ*VC1370::FRT-cat-FRT* carrying transcriptional *comEA-lacZ* fusion and mini-Tn7-*araC*-P_{BAD}-*tfoX* (Tn*tfoX*); Rif^R, $Gent^R$, Cm^R	6357	This study
*comEA-lacZ*ΔVC*1423*-Tn*tfoX*	A1552Δ*lacZ*Δ*VC1423::FRT-cat-FRT* carrying transcriptional *comEA-lacZ* fusion and mini-Tn7-*araC*-P_{BAD}-*tfoX* (Tn*tfoX*); Rif^R, $Gent^R$, Cm^R	6356	This study
*comEA-lacZ*ΔVC*1430*-Tn*tfoX*	A1552Δ*lacZ*Δ*VC1430::FRT-cat-FRT* carrying transcriptional *comEA-lacZ* fusion and mini-Tn7-*araC*-P_{BAD}-*tfoX* (Tn*tfoX*); Rif^R, $Gent^R$, Cm^R	6355	This study
*comEA-lacZ*ΔVC*1527*-Tn*tfoX*	A1552Δ*lacZ*Δ*VC1527::FRT-cat-FRT* carrying transcriptional *comEA-lacZ* fusion and mini-Tn7-*araC*-P_{BAD}-*tfoX* (Tn*tfoX*); Rif^R, $Gent^R$, Cm^R	6354	This study
*comEA-lacZ*ΔVC*1651*-Tn*tfoX*	A1552Δ*lacZ*Δ*VC1651::FRT-cat-FRT* carrying transcriptional *comEA-lacZ* fusion and mini-Tn7-*araC*-P_{BAD}-*tfoX* (Tn*tfoX*); Rif^R, $Gent^R$, Cm^R	6353	This study
*comEA-lacZ*ΔVC*1729-30*-Tn*tfoX*	A1552Δ*lacZ*Δ*VC1729-30::FRT-cat-FRT* carrying transcriptional *comEA-lacZ* fusion and mini-Tn7-*araC*-P_{BAD}-*tfoX* (Tn*tfoX*); Rif^R, $Gent^R$, Cm^R	6352	This study
*comEA-lacZ*ΔVC*1764*-Tn*tfoX*	A1552Δ*lacZ*Δ*VC1764::FRT-cat-FRT* carrying transcriptional *comEA-lacZ* fusion and mini-Tn7-*araC*-P_{BAD}-*tfoX* (Tn*tfoX*); Rif^R, $Gent^R$, Cm^R	6358	This study
*comEA-lacZ*Δ*ruvB*-Tn*tfoX*	A1552Δ*lacZ*Δ*ruvB::FRT-cat-FRT* (*VC1845*) carrying transcriptional *comEA-lacZ* fusion and mini-Tn7-*araC*-P_{BAD}-*tfoX* (Tn*tfoX*); Rif^R, $Gent^R$, Cm^R	6359	This study
*comEA-lacZ*ΔVC*2227*-Tn*tfoX*	A1552Δ*lacZ*Δ*VC2227::FRT-cat-FRT* carrying transcriptional *comEA-lacZ* fusion and mini-Tn7-*araC*-P_{BAD}-*tfoX* (Tn*tfoX*); Rif^R, $Gent^R$, Cm^R	6360	This study
*comEA-lacZ*ΔVC*2320*-Tn*tfoX*	A1552Δ*lacZ*Δ*VC2320::FRT-cat-FRT* carrying transcriptional *comEA-lacZ* fusion and mini-Tn7-*araC*-P_{BAD}-*tfoX* (Tn*tfoX*); Rif^R, $Gent^R$, Cm^R	6366	This study
*comEA-lacZ*ΔVC*2497*-Tn*tfoX*	A1552Δ*lacZ*Δ*VC2497::FRT-cat-FRT* carrying transcriptional *comEA-lacZ* fusion and mini-Tn7-*araC*-P_{BAD}-*tfoX* (Tn*tfoX*); Rif^R, $Gent^R$, Cm^R	6361	This study
*comEA-lacZ*ΔVC*2757*-Tn*tfoX*	A1552Δ*lacZ*Δ*VC2757::FRT-cat-FRT* carrying transcriptional *comEA-lacZ* fusion and mini-Tn7-*araC*-P_{BAD}-*tfoX* (Tn*tfoX*); Rif^R, $Gent^R$, Cm^R	6362	This study
*comEA-lacZ*ΔVC*A0536*-Tn*tfoX*	A1552Δ*lacZ*Δ*VCA0536::FRT-cat-FRT* carrying transcriptional *comEA-lacZ* fusion and mini-Tn7-*araC*-P_{BAD}-*tfoX* (Tn*tfoX*); Rif^R, $Gent^R$, Cm^R	6363	This study

*comEA-lacZ*Δ*VCA0571*-Tn*tfoX*	A1552Δ*lacZ*Δ*VCA0571::FRT-cat-FRT* carrying transcriptional *comEA-lacZ* fusion and mini-Tn7-*araC*-P_{BAD}-*tfoX* (Tn*tfoX*)*;* Rif^R, $Gent^R$, Cm^R	6364	This study
*comEA-lacZ*Δ*VCA0691*-Tn*tfoX*	A1552Δ*lacZ*Δ*VCA0691::FRT-cat-FRT* carrying transcriptional *comEA-lacZ* fusion and mini-Tn7-*araC*-P_{BAD}-*tfoX* (Tn*tfoX*)*;* Rif^R, $Gent^R$, Cm^R	6365	This study
*comEA-lacZ*Δ*VCA0773*-Tn*tfoX*	A1552Δ*lacZ*Δ*VCA0773::FRT-cat-FRT* carrying transcriptional *comEA-lacZ* fusion and mini-Tn7-*araC*-P_{BAD}-*tfoX* (Tn*tfoX*)*;* Rif^R, $Gent^R$, Cm^R	6341	This study
*comEA-lacZ*Δ*VCA0781*-Tn*tfoX*	A1552Δ*lacZ*Δ*VCA0781::FRT-cat-FRT* carrying transcriptional *comEA-lacZ* fusion and mini-Tn7-*araC*-P_{BAD}-*tfoX* (Tn*tfoX*)*;* Rif^R, $Gent^R$, Cm^R	6342	This study
*comEA-lacZ*Δ*vesA*-Tn*tfoX*	A1552Δ*lacZ*Δ*vesA::FRT-cat-FRT* (*VCA0803*) carrying transcriptional *comEA-lacZ* fusion and mini-Tn7-*araC*-P_{BAD}-*tfoX* (Tn*tfoX*)*;* Rif^R, $Gent^R$, Cm^R	6343	This study
*comEA-lacZ*Δ*VCA1012*-Tn*tfoX*	A1552Δ*lacZ*Δ*VCA1012::FRT-cat-FRT* carrying transcriptional *comEA-lacZ* fusion and mini-Tn7-*araC*-P_{BAD}-*tfoX* (Tn*tfoX*)*;* Rif^R, $Gent^R$, Cm^R	6344	This study
*comEA-lacZ*Δ*VCA1048*-Tn*tfoX*	A1552Δ*lacZ*Δ*VCA1048::FRT-cat-FRT* carrying transcriptional *comEA-lacZ* fusion and mini-Tn7-*araC*-P_{BAD}-*tfoX* (Tn*tfoX*)*;* Rif^R, $Gent^R$, Cm^R	6345	This study
*comEA-lacZ*Δ*trmY*-Tn*tfoX*	A1552Δ*lacZ*Δ*trmY::FRT-cat-FRT* (*VCA1059*) carrying transcriptional *comEA-lacZ* fusion and mini-Tn7-*araC*-P_{BAD}-*tfoX* (Tn*tfoX*)*;* Rif^R, $Gent^R$, Cm^R	6346	This study
ΔVcr2	A1552ΔVcr2*::FRT-cat-FRT*; Rif^R, Cm^R	6422	This study
*comEA-lacZ*ΔVcr2-Tn*tfoX*	A1552ΔVcr2*::FRT-cat-FRT* carrying transcriptional *comEA-lacZ* fusion and mini-Tn7-*araC*-P_{BAD}-*tfoX* (Tn*tfoX*)*;* Rif^R, $Gent^R$, Cm^R	6423	This study
ΔVcr83	A1552ΔVcr83*::FRT-cat-FRT*; Rif^R, Cm^R	6424	This study
*comEA-lacZ*ΔVcr83-Tn*tfoX*	A1552ΔVcr83*::FRT-cat-FRT* carrying transcriptional *comEA-lacZ* fusion and mini-Tn7-*araC*-P_{BAD}-*tfoX* (Tn*tfoX*)*;* Rif^R, $Gent^R$, Cm^R	6425	This study
Δ*hfq*-Tn*tfoX*	A1552Δ*hfq*::FRT carrying mini-Tn7-*araC*-P_{BAD}-*tfoX* (Tn*tfoX*)*;* Rif^R, $Gent^R$	3949	This study
Δ*VCA0199*	A1552Δ*VCA0199::FRT*, Rif^R	6316	This study
Δ*VC1746*	A1552Δ*VC1746::FRT;* Rif^R	6317	This study
Δ*VCA0767*	A1552Δ*VCA0767::FRT;* Rif^R	6318	This study
*comEA-lacZ*Δ*VCA0199*-Tn*tfoX*	A1552Δ*lacZ*Δ*VCA0199::FRT-aph-FRT* carrying transcriptional *comEA-lacZ* fusion and mini-Tn7-*araC*-P_{BAD}-*tfoX* (Tn*tfoX*)*;* Rif^R, $Gent^R$, Kan^R	6367	This study
*comEA-lacZ*Δ*VC1746*-Tn*tfoX*	A1552Δ*lacZ* Δ*VC1746::FRT-cat-FRT* carrying transcriptional *comEA-lacZ* fusion and mini-Tn7-*araC*-P_{BAD}-*tfoX* (Tn*tfoX*)*;* Rif^R, $Gent^R$, Cm^R	6368	This study
*comEA-lacZ*Δ*VCA0767*-Tn*tfoX*	A1552Δ*lacZ* Δ*VCA0767::FRT-cat-FRT* carrying transcriptional *comEA-lacZ* fusion and mini-Tn7-*araC*-P_{BAD}-*tfoX* (Tn*tfoX*)*;* Rif^R, $Gent^R$, Cm^R	6369	This study

Δ*gidA*-Tn*tfoX*	A1552Δ*gidA::FRT* (*VC2775*) carrying mini-Tn7-*araC*-P_{BAD}-*tfoX* (Tn*tfoX*)*;* Rif^R, $Gent^R$	6319	This study
Δ*trmE*-Tn*tfoX*	A1552Δ*trmE::FRT* (*VC0003*) carrying mini-Tn7-*araC*-P_{BAD}-*tfoX* (Tn*tfoX*)*;* Rif^R, $Gent^R$	6320	This study
Δ*miaA*-Tn*tfoX*	A1552Δ*miaA::FRT* (*VC0346*) carrying mini-Tn7-*araC*-P_{BAD}-*tfoX* (Tn*tfoX*)*;* Rif^R, $Gent^R$	6321	This study
Δ*VC0539*-Tn*tfoX*	A1552Δ*VC0539::FRT* carrying mini-Tn7-*araC*-P_{BAD}-*tfoX* (Tn*tfoX*)*;* Rif^R, $Gent^R$	6322	This study
Δ*VC0767*-Tn*tfoX*	A1552Δ*VC0767::FRT* carrying mini-Tn7-*araC*-P_{BAD}-*tfoX* (Tn*tfoX*)*;* Rif^R, $Gent^R$	6323	This study
ΔVC*2067*-Tn*tfoX*	A1552Δ*VC2067::FRT* carrying mini-Tn7-*araC*-P_{BAD}-*tfoX* (Tn*tfoX*)*;* Rif^R, $Gent^R$	6324	This study
Δ*flgD*-Tn*tfoX*	A1552Δ*flD::FRT* (*VC2198*) carrying mini-Tn7-*araC*-P_{BAD}-*tfoX* (Tn*tfoX*)*;* Rif^R, $Gent^R$	6325	This study
Δ*flaA*-Tn*tfoX*	A1552Δ*flaA::FRT* (*VC2188*) carrying mini-Tn7-*araC*-P_{BAD}-*tfoX* (Tn*tfoX*)*;* Rif^R, $Gent^R$	2000	This study
Δ*pomB*-Tn*tfoX*	A1552Δ*pomB::FRT* (*VC0893*) carrying mini-Tn7-*araC*-P_{BAD}-*tfoX* (Tn*tfoX*)*;* Rif^R, $Gent^R$	5872	This study
Δ*hapR*-Tn*tfoX*	A1552Δ*hapR* carrying mini-Tn7-*araC*-P_{BAD}-*tfoX* (Tn*tfoX*)*;* Rif^R, $Gent^R$	1627	(12)
i*tfoX*Δ*flgD*	A1552Δ*lacZ*::i*tfoX* Δ*flgD::FRT*; Rif^R	6326	This study
i*tfoX*Δ*flgD*-Tn*qstR*	A1552Δ*lacZ*::i*tfoX* Δ*flgD::FRT* carrying mini-Tn7-*araC*-P_{BAD}-*qstR* (Tn*qstR*)*;* Rif^R, $Gent^R$	6327	This study
i*tfoX*Δ*VC2067*	A1552Δ*lacZ*::i*tfoX* Δ*VC2067::FRT*; Rif^R	6328	This study
i*tfoX*Δ*VC2067*-Tn*qstR*	A1552Δ*lacZ*::i*tfoX*Δ*VC2067::FRT* carrying mini-Tn7-*araC*-P_{BAD}-*qstR* (Tn*qstR*)*;* Rif^R, $Gent^R$	6329	This study
*comEA-lacZ*Δ*gidA*-Tn*tfoX*	A1552Δ*lacZ*Δ*gidA::FRT-cat-FRT* (*VC2775*) carrying transcriptional *comEA-lacZ* fusion and mini-Tn7-*araC*-P_{BAD}-*tfoX* (Tn*tfoX*)*;* Rif^R, $Gent^R$, Cm^R	6370	This study
*comEA-lacZ*Δ*trmE*-Tn*tfoX*	A1552Δ*lacZ*Δ*trmE::FRT-cat-FRT* (*VC0003*) carrying transcriptional *comEA-lacZ* fusion and mini-Tn7-*araC*-P_{BAD}-*tfoX* (Tn*tfoX*)*;* Rif^R, $Gent^R$, Cm^R	6371	This study
*comEA-lacZ*Δ*miaA*-Tn*tfoX*	A1552Δ*lacZ*Δ*miaA::FRT-cat-FRT* (*VC0346*) carrying transcriptional *comEA-lacZ* fusion and mini-Tn7-*araC*-P_{BAD}-*tfoX* (Tn*tfoX*)*;* Rif^R, $Gent^R$, Cm^R	6372	This study
comEA-lacZ Δ*VC0539*-Tn*tfoX*	A1552Δ*lacZ*Δ*VC0539::FRT-cat-FRT* carrying transcriptional *comEA-lacZ* fusion and mini-Tn7-*araC*-P_{BAD}-*tfoX* (Tn*tfoX*)*;* Rif^R, $Gent^R$, Cm^R	6373	This study
*comEA-lacZ*Δ*VC0767*-Tn*tfoX*	A1552Δ*lacZ*Δ*VC0767::FRT-cat-FRT* carrying transcriptional *comEA-lacZ* fusion and mini-Tn7-*araC*-P_{BAD}-*tfoX* (Tn*tfoX*)*;* Rif^R, $Gent^R$, Cm^R	6375	This study
*comEA-lacZ*ΔVC*2067*-Tn*tfoX*	A1552Δ*lacZ*Δ*VC2067::FRT-cat-FRT* carrying transcriptional *comEA-lacZ* fusion and mini-Tn7-*araC*-P_{BAD}-*tfoX* (Tn*tfoX*)*;* Rif^R, $Gent^R$, Cm^R	6374	This study

*comEA-lacZ*Δ*flgD*-Tn*tfoX*	A1552Δ*lacZ*Δ*flgD::FRT-cat-FRT* (*VC2198*) carrying transcriptional *comEA-lacZ* fusion and mini-Tn7-*araC*-P_{BAD}-*tfoX* (Tn*tfoX*)*;* Rif^R, $Gent^R$, Cm^R	6376	This study
Escherichia coli			
TOP10	F– *mcrA* Δ(*mrr-hsd*RMS-*mcr*BC) Φ80*lacZ*ΔM15 Δ*lac*X74 *recA1 araD*139 Δ(*ara leu*) 7697 *galU galK rpsL* (Str^R) *endA*1 *nupG*	741	Invitrogen
DH5α	F^- ϕ80*lacZ*ΔM15 Δ(*lacZYA-argF*) U169 *deoR recA1 endA1 hsd*R17 (rk-, mk+) *phoA supE*44 λ- thi-1 *gyrA*96 *relA*1	736	Invitrogen
XL10-Gold	Tet^RΔ(*mcrA*)183 Δ(*mcrCB-hsdSMR-mrr*)173 *endA1 supE*44 *thi*-1 *recA*1 *gyrA*96 *relA*1 *lac* Hte [F´ *proAB lacI*^{q}ZΔM15 Tn10 (Tet^R) Amy Cam^R]	734	Stratagene
S17-1λpir	Tp^R Sm^R *recA thi pro hsdR*-M+ RP4:2-Tc:Mu:Kan^R Tn7 (λpir)	648	(21)
MFD*pir* (UGB 3093)	MG1655 RP4-2-Tc::[Mu1::*aac*(3)IV-Δ*aphA*-Δ*nic35*-ΔMu2::zeo	4662	(22)
BTH101	F^-, *cya*-99, *araD*139, *galE*15, *galK*16, *rpsL*1 (Str^R), *hsdR*2, *mcrA*1, *mcrB*1.	6221	Euromedex
BL21 (DE3)	*E. coli* B F^- *dcm ompT hsdS*(r_B^- m_B^-) *gal* λ(DE3)	978	Agilent
**Plasmids**			
pBAD/Myc-HisA	pBR322-derived expression vector; *araBAD* promoter (P_{BAD}); Amp^R	24	Invitrogen
pBAD-*qstR*-N-strep	*qstR* gene preceded by sequence encoding Strep-tagII® cloned into pBAD/Myc-HisA; arabinose inducible; Amp^R	2745	This study
pBAD-*qstR*[P129T]-N-strep	qstR[P129T] preceded by sequence encoding Strep-tagII® in pBAD/Myc-HisA vector obtained by inverse PCR; arabinose inducible; Amp^R	6382	This study
pBAD-*qstR*[P129A]-N-strep	qstR[P129A] preceded by sequence encoding Strep-tagII® in pBAD/Myc-HisA vector obtained by inverse PCR; arabinose inducible; Amp^R	6383	This study
pBAD-*qstR*[R130A]-N-strep	qstR[R130A] preceded by sequence encoding Strep-tagII® in pBAD/Myc-HisA vector obtained by inverse PCR; arabinose inducible; Amp^R	6384	This study
pBAD-*qstR*[L137A]-N-strep	qstR[L137A] preceded by sequence encoding Strep-tagII® in pBAD/Myc-HisA vector obtained by inverse PCR; arabinose inducible; Amp^R	6385	This study
pBAD-*qstR*[R141A]-N-strep	qstR[R141A] preceded by sequence encoding Strep-tagII® in pBAD/Myc-HisA vector obtained by inverse PCR; arabinose inducible; Amp^R	6386	This study

pBR-FRT-Kan-FRT2	pBR322 derivative containing improved FRT-*aph*-FRT cassette, used as template for TransFLP; Amp^R, Kan^R	3782	(16)
pBR-FRT-Cat-FRT2	pBR322 derivative containing improved FRT-*cat*-FRT cassette, used as template for TransFLP; Amp^R, Cam^R	3783	(16)
pBR-flp	pBR322 derivative containing FLP+, λ cI857+, λ pR from pCP20 integrated into the *EcoRV* site of pBR322, used for FLP recombination; Amp^R	1203	(23)
pFRT-aph-pheS*	pBR322 derivative containing improved FRT-*aph*-FRT cassette, as well as *pheS**[A294G/T251A], used as template for Trans2; Amp^R, Kan^R	4591	(24)
pUX-BF13	pUX-BF13 - oriR6K, helper plasmid with Tn7 transposition function; Amp^R	457	(25)
pGP704-mTn	pGP704 with empty mini-Tn7 (Tn); Amp^R, $Gent^R$	1997	(26)
pGP704-Tn*tfoX*	pGP704 with mini-Tn7 carrying *araC* and P_{BAD}-*tfoX*; Amp^R, $Gent^R$ (Tn*tfoX*); Amp^R, $Gent^R$	1624	(12)
pGP704-Tn*vdcA*	pGP704 with mini-Tn7 carrying *araC* and P_{BAD}-*vdcA*; Amp^R, $Gent^R$ (Tn*vdcA*); Amp^R, $Gent^R$	2943	(16)
pGP704-Tn*cdpA*	pGP704 with mini-Tn7 carrying *araC* and P_{BAD}-*cdpA*; Amp^R, $Gent^R$ (Tn*cdpA*); Amp^R, $Gent^R$	2944	(16)
pGP704-Tn*qstR*	pGP704 with mini-Tn7 carrying *araC* and P_{BAD}-*qstR*; Amp^R, $Gent^R$ (Tn*qstR*); Amp^R, $Gent^R$	5494	This study
pGP704-Tn*qstR*[L137A]	pGP704 with mini-Tn7 carrying *araC* and P_{BAD}-*qstR*[L137A]; Amp^R, $Gent^R$ (Tn*qstR*[L137A]); Amp^R, $Gent^R$	6387	This study
pGP704-28-SacB-Δ*qstR*	pGP704-Sac28 with a gene fragment resulting in a 402-bp deletion (incl. stop codon) within *qstR*; Amp^R	1118	(14)
pGP704-28-SacB-ΔcomM	pGP704-Sac28 with a gene fragment resulting in deletion within *comM;* Amp^R	1079	This study
pGP704-28-SacB-Δ*ligA2*	pGP704-Sac28 with a gene fragment resulting in deletion within *ligA2;* Amp^R	1140	This study
pGP704-28-SacB-Δ*VC0542*	pGP704-Sac28 with a gene fragment resulting in deletion within *VC0542;* Amp^R	1098	This study
pGP704-28-SacB-Δ*hapR*	pGP704-Sac28 with a gene fragment resulting in deletion within *hapR;* Amp^R	1038	(19)
pGP704-28-SacB-Δ*VC0470*	pGP704-Sac28 with a gene fragment resulting in deletion within *VC0470* (*dns*)*;* Amp^R	1093	(18)
pSC189	Plasmid for the deliver of *mariner*-based transposon; Amp^R, Kan^R	6089	Gift from F. Yildiz; (1)
pUT18	pUT18; BACTH C-terminal T18 fusions, Amp^R	6222	Euromedex

pUT18C	pUT18C; BACTH N-terminal T18 fusions, Amp^R	6223	Euromedex
pUT18C-zip	T18-leucine zipper from yeast GCN4; Amp^R	6224	Euromedex
pKT25	pKT25; BACTH N-terminal T25 fusions, Kan^R	6225	Euromedex
pKNT25	pKNT25; BACTH C-terminal T25 fusions, Kan^R	6226	Euromedex
pKT25-zip	T25-leucine zipper from yeast GCN4; Kan^R	6227	Euromedex
pUT18-*qstR*	plasmid encoding QstR(T18); Amp^R	6388	This study
pUT18C-*qstR*	plasmid encoding (T18)QstR; Amp^R	6389	This study
pKT25-*qstR*	plasmid encoding (T25)QstR; Kan^R	6390	This study
pKNT25-*qstR*	plasmid encoding QstR(T25); Kan^R	6391	This study
pUT18-*qstR*[P129T]	plasmid encoding QstR[P129T](T18); Amp^R	6392	This study
pUT18-*qstR*[P129A]	plasmid encoding QstR[P129A](T18); Amp^R	6393	This study
pUT18-*qstR*[R130A]	plasmid encoding QstR[R130A](T18); Amp^R	6394	This study
pUT18-*qstR*[L137A]	plasmid encoding QstR[L137A](T18); Amp^R	6396	This study
pUT18-*qstR*[R141A]	plasmid encoding QstR[R141A](T18); Amp^R	6395	This study
pKNT25-*qstR*[P129T]	plasmid encoding QstR[P129T](T25); Kan^R	6397	This study
pKNT25-*qstR*[P129A]	plasmid encoding QstR[P129A](T25); Kan^R	6398	This study
pKNT25-*qstR*[R130A]	plasmid encoding QstR[R130A](T25); Kan^R	6399	This study
pKNT25-*qstR*[L137A]	plasmid encoding QstR[L137A](T25); Kan^R	6401	This study
pKNT25-*qstR*[R141A]	plasmid encoding QstR[R141A](T25); Kan^R	6400	This study

VC locus tag numbers according to Heidelberg *et al.*, 2000 (27).

Table S2. QstR dependent peaks identified in the ChIP-seq experiment.

Peak ID	Chromosome*	Start	End	Peak Score	Distance to TSS#	*Nearest TSS#*	Gene potentially regulated
1	NC_002505	29919	30019	43462.2	-100	*VC0032*	***comM (VC0032)***
2	NC_002505	499418	499518	34996.5	-36	*VC0470*	***dns (VC0470)***
3	NC_002505	2681903	2682003	21431.7	1220	*VC2497*	***VC2497***
4	NC_002505	1188140	1188240	20644.4	-201	*VC1120*	***VC1120***
5	NC_002505	223428	223528	13998.6	24	*VC0216*	***VC2016***
6	NC_002505	2893822	2893922	11938.9	20	*VC2718*	***comF (VC2719)***
7	NC_002505	1014426	1014526	11461.2	-259	*VC0949*	***rodA (VC0949)***
8	NC_002505	1867102	1867202	10664.2	-602	*VC1727*	***VC1729, VC1730***
9	NC_002506	116334	116434	8806.4	-462	*VCA0107*	***vipA (VCA0107)***
10	NC_002506	474233	474333	8361.7	-245	*VCA0536*	***VCA0536***
11	NC_002505	2793096	2793196	7838.5	-79	*VC2621*	***xds (VC2621)***
12	NC_002506	746353	746453	6858.9	-56	*VCA0803*	***vesA (VCA0803)***
13	NC_002505	2026448	2026548	6408	-580	*VC1879*	***comEC (VC1879)***
14	NC_002505	1860554	1860654	6257.6	-286	*VC1722*	***tfoY (VC1722)***
15	NC_002505	225092	225192	6094.6	-118	*VC0217*	***radC (VC0217)***
16	NC_002505	1055786	1055886	5205.6	789	*VC0991*	***asnB (VC0991)***
17	NC_002505	1655807	1655907	3707.1	-102	*VC1542*	***ligA2 (VC1542)***
18	NC_002506	509039	509139	3579.1	-102	*VCA0571*	***VCA0571***
19	NC_002505	1858513	1858613	3348.4	111	*VC1720*	***VC1720***
20	NC_002505	2596898	2596998	3250.4	-203	*VC2423*	***pilA (VC2423)***
21	NC_002506	962459	962559	3180	70	*VCA1012*	***VCA1012***
22	NC_002506	630515	630615	2990.9	-503	*VCA0691*	***VCA0691***
23	NC_002505	1520036	1520136	2883.2	247	*VC1422*	***VC1423***
24	NC_002505	422391	422491	2850.8	-119	*VC0396*	***qstR (VC0396)***
25	NC_002505	1279235	1279335	2744	250	*VC1203*	***VC1203***
26	NC_002505	2942069	2942169	2743	-8	*VC2757*	***VC2757***
27	NC_002505	44748	44848	2676.1	10	*VC0046*	***tsaP (VC0047)***
28	NC_002505	1905612	1905712	2665.3	83	*VC1764*	***VC1764***
29	NC_002505	963786	963886	2587.2	150	*VC0903*	***VC0903***
30	NC_002505	1780458	1780558	2427.8	461	*VC1651*	***VC1651***
31	NC_002505	2937010	2937110	2416.9	2247	*VC2753*	
32	NC_002505	1643003	1643103	2322.7	1093	*VC1530*	***VC1527***
33	NC_002506	730580	730680	2009.4	-336	*VCA0786*	***VCA0786***
34	NC_002505	5230	5330	1891.9	328	*VC0008*	***VC0008***
35	NC_002505	576738	576838	1829.9	264	*VC0545*	***alaS (VC0545)***
36	NC_002506	833625	833725	1826.5	178	*VCA0882*	***VCA0882***
37	NC_002505	800943	801043	1801.9	-208	*VC0748*	***VC0748***
38	NC_002505	2472311	2472411	1711.8	-102	*VC2320*	***VC2320***
39	NC_002505	1873597	1873697	1671.1	55	*VC1737*	***infA (VC1737)***
40	NC_002505	870392	870492	1550.2	-51	*VC0813*	***VC0813***
41	NC_002505	180805	180905	1481	436	*VC0179*	***VC0179***
42	NC_002506	716241	716341	1422.4	1005	*VCA0773*	***VCA0773***
43	NC_002505	268976	269076	1370.4	-42	*VC0264*	***VC0264***
44	NC_002505	1527906	1528006	1316.8	-84	*VC1430*	***VC1430***
45	NC_002505	1460085	1460185	1315.2	382	*VC1370*	***VC1370***
46	NC_002505	2381733	2381833	1284.4	198	*VC2227*	***purN (VC2227)***
47	NC_002505	624040	624140	1243.9	-345	*VC0596*	***dksA (VC0596)***
48	NC_002505	553620	553720	1223.5	475	*VC0518*	***dnaG (VC0518)***
49	NC_002506	1009892	1009992	1201.2	-60	*VCA1059*	***VCA1059***
50	NC_002506	724279	724379	1057.3	-947	*VCA0780*	***VCA0781***
51	NC_002506	998707	998807	1001.6	-72	*VCA1048*	***VCA1048***
52	NC_002505	1985563	1985663	863.9	287	*VC1845*	***ruvB (VC1845)***

* Chromosome I (accession number NC_002505), chromosome II (accession number NC_002506); (27).

# Translational start site as predicted by Heidelberg *et al.*, 2000 (27).

Table S3. Homer *de novo* motif analysis of the QstR-dependent ChIP-seq peak sequences.

Rank	Motif	p-value	q-value/FDR	% of Targets[#]	% of Background[#]	Nearest TSS[a / b]
1	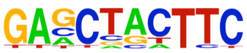	1e-10	0.030	25.00%	1.92%	*VC1727*, ***ligA2***, ***qstR***, *VC1651*, *VC2753*, *VC1530*, *VCA0882*, *VC2320*, *VC0813*, *VCA0773*, *VC1370*, *dksA*, *ruvB*
2	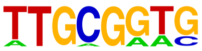	1e-8	0.090	30.77%	5.09%	*VC1120*, *VC1727*, *VCA0536*, *vesA*, ***comEC***, *radC*, *asnB*, *pilA*, *VC1422*, ***qstR***, *VC1764*, *alaS*, *infA*, *VCA0773*, *VC1430*, *dksA*
3*	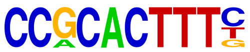	1e-8	0.290	9.62%	0.12%	*rodA*, *asnB*, *alaS*, *VCA0773*, *VCA1059*
4*	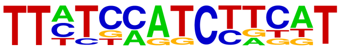	1e-7	1.000	11.54%	0.30%	***comF***, *VC2757*, *VCA0786*, *VC0008*, *VC0813*, *VCA1048*
5*	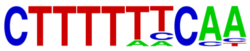	1e-7	0.367	13.46%	0.62%	***comM***, ***vipA***, ***comEC***, *VC1651*, *VCA0786*, *VC0008*, *ruvB*
6*	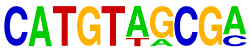	1e-7	0.367	7.69%	0.06%	***comM***, *VCA0786*, *VC1430*, *VC1370*
7*	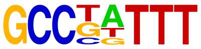	1e-6	0.49	25.00%	4.29%	***vipA***, *VCA0571*, *VC1720*, ***qstR***, *VC2757*, *VC0903*, *VC1651*, *VC0179*, *purN*, *dksA*, *VCA1059*, *VCA1048*, *ruvB*
8*	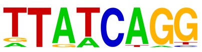	1e-5	0.49	15.38%	1.50%	***dns***, *VC2497*, *VC1727*, ***vipA***, *pilA*, *VCA0691*, ***qstR***, *VC0748*
9*	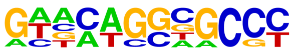	1e-5	1.000	9.62%	0.35%	*VC1422*, ***qstR***, *VC2757*, *VC1651*, *purN*
10*	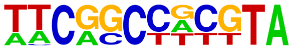	1e-5	1.000	9.62%	0.43%	*VC0216*, *vesA*, ***ligA2***, *VC0008*, *VC1370*

* Possible false positive.

# Total target sequences = 52, Total Background Sequences = 34083.

[a] Translational start site as predicted by Heidelberg *et al.*, 2000 (27).

[b] Genes regulated in a QstR-dependent manner are shown in bold.

Table S4. Genes identified in the transposon mutagenesis screens

1 (> 20 000 colonies screened)

No of hits	color	Tn location	Comment (reference)
20	white	*comEA-lacZ* region	*comEA-lacZ* reporter
9	white	Tn*tfoX*	inducible *tfoX*
7	white	*VC0396 (qstR)*	required for *comEA* expression (14)
5	white	*VC0122 (cyaA)*	required for *comEA* expression (28)
4	white	*VC0583 (hapR)*	required for *comEA* expression (12,19)
3	white	*VC2677 (cytR)*	required for *comEA* expression (29)
5	faint blue	*VC2775 (gidA)*	tRNA uridine 5-carboxymethylaminomethyl modification protein GidA
4	faint blue	*VC0003 (mnmE, trmE)*	tRNA modification GTPase
2	faint blue	*VC0346 (miaA)*	tRNA delta(2)-isopentenylpyrophosphate transferase
1	white	*VCA0518*	PTS system fructose-specific transporter subunitIIA/HPr protein
1	faint blue	*VC2267 (nusB)*	transcription antitermination protein
1	faint blue	*VC0539*	sulfate ABC transporter
1	faint blue	*VC0541*	sulfate ABC transporter
1	faint blue	*VC0709 (rluD)*	23S rRNA pseudouridine synthase D
1	faint blue	*VC2067*	MinD like protein
1	faint blue	*VC2198 (flgD)*	flagellar basal body rod modification protein
1	faint blue	*VC2026*	hypothetical protein
1	faint blue	*VC2629 (aroK)*	shikimate kinase I
1	faint blue	*VC2044*	perosamine synthase
1	faint blue	*VC0556*	glutamate-cysteine ligase
1	faint blue	*VC0894*	thiamine biosynthesis protein
1	faint blue	*VC2649 (cysE)*	serine acetyltransferase

2 (> 60 000 colonies screened)

No of hits	color	Tn location	comment
61	white	*comEA-lacZ* region	*comEA-lacZ* reporter
27	white	Tn*tfoX*	inducible *tfoX*
21	white	*VC0396 (qstR)*	required for *comEA* expression (14)
10	white	*VC0583 (hapR)*	required for *comEA* expression (12,19)
5	white	*VC2677 (cytR)*	required for *comEA* expression (29)
4	white	*VC0122 (cyaA)*	required for *comEA* expression (28)
2	white	*VC1213 (varA)*	response regulator
4	faint blue	*VC2067*	MinD like protein
2	faint blue	*VC0767 (guaB)*	inosine 5'-monophosphate dehydrogenase
1	white	*VC2012*	sodium dependent transporter
1	white	*VC2047*	upstream, short chain dehydrogenase
1	white	VC0251	acyl proteinsynthase/acyl-CoA reductase
1	faint blue	*VC2465 (rseB)*	negative regulator of sigma E
1	faint blue	*VC2198 (flgD)*	flagellar basal body rod modification protein
1	faint blue	*VC2548*	hypothetical protein
1	faint blue	*VC0003 (mnmE, trmE)*	tRNA modification GTPase
1	faint blue	*VC0346 (miaA)*	tRNA delta(2)-isopentenylpyrophosphate transferase
1	faint blue	*VCA0459* (upstream)	hypothetical protein

VC locus tag numbers according to Heidelberg *et al.*, 2000 (27).

Table S5. Proteins bound to the *comEA* probe in the DNA affinity pull down experiment

500 mM NaCl fractions							
				**Total spectra* (unique peptides)**			
**Position**	**Protein**	**Size**	**Function**	***comEA* probe Tn*tfoX* lysate**	***comEA* probe Tn*tfoX* Δ*qstR* lysate**	***qstR* probe Tn*tfoX* lysate**	***gyrA* probe Tn*tfoX* lysate**
1	VC0329	155 kDa	RpoC, DNA-directed RNA polymerase subunit beta'	84 (63)	73 (59)	97 (70)	109 (76)
2	VCA0940	28 kDa	Transcriptional regulator, DeoR family	75 (30)	79 (34)	63 (24)	97 (30)
3	VC0328	149 kDa	RpoB, DNA-directed RNA polymerase subunit beta	72 (56)	80 (59)	103 (69)	80 (55)
**4**	**VCA0199**	**74 kDa**	**Putative uncharacterized protein**	**65 (46)**	**51 (39)**	**0**	**2 (2)**
5	VC0766	51 kDa	XseA, Exodeoxyribonuclease 7 large subunit	41 (24)	42 (29)	27 (24)	28 (21)
6	VCA0975	89 kDa	ATP-dependent protease LA-related protein	38 (27)	38 (29)	18 (15)	18 (14)
7	VC1130	15 kDa	DNA-binding protein H-NS	35 (11)	71 (19)	185 (26)	16 (10)
8	VCA1023	70 kDa	Putative uncharacterized protein	31 (27)	33 (28)	26 (23)	27 (23)
9	VC0723	82 kDa	Ppk, Polyphosphate kinase	29 (24)	30 (26)	34 (31)	45 (36)
10	VC2501	55 kDa	PepA, Cytosol aminopeptidase	27 (22)	27 (23)	22 (19)	46 (30)
11	VC1148	32 kDa	Putative uncharacterized protein VC1148	27 (18)	23 (16)	6 (6)	64 (24)
12	VC2096	20 kDa	SeqA protein	26 (12)	33 (13)	56 (16)	47 (15)
13	VC2430	85 kDa	Topoisomerase IV, subunit A	26 (21)	24 (21)	26 (21)	23 (20)
14	VC2571	36 kDa	RpoA, DNA-directed RNA polymerase subunit alpha	23 (17)	33 (20)	35 (20)	30 (19)
15	VC1730	98 kDa	DNA topoisomerase 1	21 (18)	23 (20)	17 (14)	18 (15)
16	VC2740	35 kDa	Putative uncharacterized protein	18 (14)	14 (12)	13 (10)	11 (10)
17	VC0718	34 kDa	Recombination-associated protein RdgC	17 (15)	17 (16)	7 (7)	8 (7)
18	VC2590	26 kDa	RpsC, 30S ribosomal protein S3	16 (11)	13 (9)	18 (13)	14 (10)
19	VC0517	71 kDa	RNA polymerase sigma factor RpoD	15 (11)	8 (7)	33 (27)	26 (22)
20	VCA0002	75 kDa	Putative uncharacterized protein	12 (12)	7 (7)	6 (6)	5 (5)
21	VC2613	33 kDa	Phosphoribulokinase	12 (8)	13 (9)	13 (9)	14 (9)
22	VC2282	40 kDa	Putative uncharacterized protein	12 (11)	9 (8)	14 (12)	14 (11)
23	VC0394	104 kDa	UvrABC system protein A	11 (10)	13 (13)	8 (8)	10 (10)
24	VC2506	109 kDa	RNA polymerase-associated protein RapA	10 (10)	11 (11)	7 (7)	9 (9)
25	VCA0453	18 kDa	Putative uncharacterized protein	9 (7)	10 (8)	10 (8)	2 (2)
26	VC2039	38 kDa	Nucleoid-associated protein NdpA	8 (8)	12 (12)	10 (9)	6 (6)
27	VC0273	9 kDa	HupA, DNA-binding protein HU-alpha	8 (5)	11 (4)	26 (7)	8 (4)
28	VC2249	17 kDa	(3R)-hydroxymyristoyl-[acyl-carrier-protein] dehydratase, fatty acid biosynthetic process	7 (7)	10 (9)	8 (7)	10 (8)
29	VC1758	47 kDa	Integrase, phage family	7 (6)	4 (4)	35 (23)	11 (9)
**30**	**VC1746**	**24 kDa**	**Transcriptional regulator, TetR family**	**7 (6)**	**4 (4)**	**1 (1)**	**0**
31	VCA0723	46 kDa	3-hydroxy-3-methylglutaryl CoA reductase	6 (4)	9 (5)	7 (5)	9 (7)
32	VC0397	20 kDa	SSB, Single-stranded DNA-binding protein	5 (4)	3 (3)	3 (3)	7 (5)
**33**	**VCA0767**	**29 kDa**	**Transcriptional regulator, TetR family**	**5 (4)**	**4 (3)**	**0**	**0**
34	VC0321/ VC0362	43 kDa	Elongation factor Tu-A/ Elongation factor Tu-B	4 (4)	7 (7)	5 (5)	6 (6)
35	VC2709	10 kDa	RpoZ, DNA-directed RNA polymerase subunit omega	4 (3)	5 (3)	3 (3)	5 (4)
36	VC2373	167 kDa	Glutamate synthase, large subunit	4 (4)	0	0	0

VC locus tag numbers according to Heidelberg *et al.*, 2000 (27).

* >4 total spectra for the *comEA* probe incubated with Tn*tfoX* strain lysate.

Supplementary references

1. Chiang, S.L. and Rubin, E.J. (2002) Construction of a mariner-based transposon for epitope-tagging and genomic targeting. *Gene*, **296**, 179-185.
2. Lenz, D.H., Miller, M.B., Zhu, J., Kulkarni, R.V. and Bassler, B.L. (2005) CsrA and three redundant small RNAs regulate quorum sensing in *Vibrio cholerae*. *Mol Microbiol*, **58**, 1186-1202.
3. Yim, L., Martinez-Vicente, M., Villarroya, M., Aguado, C., Knecht, E. and Armengod, M.E. (2003) The GTPase activity and C-terminal cysteine of the *Escherichia coli* MnmE protein are essential for its tRNA modifying function. *J Biol Chem*, **278**, 28378-28387.
4. Yim, L., Moukadiri, I., Bjork, G.R. and Armengod, M.E. (2006) Further insights into the tRNA modification process controlled by proteins MnmE and GidA of *Escherichia coli*. *Nucleic Acids Res*, **34**, 5892-5905.
5. Nakayashiki, T. and Inokuchi, H. (1998) Novel temperature-sensitive mutants of *Escherichia coli* that are unable to grow in the absence of wild-type tRNA6Leu. *J Bacteriol*, **180**, 2931-2935.
6. Bregeon, D., Colot, V., Radman, M. and Taddei, F. (2001) Translational misreading: a tRNA modification counteracts a +2 ribosomal frameshift. *Genes Dev*, **15**, 2295-2306.
7. Wang, Q., Millet, Y.A., Chao, M.C., Sasabe, J., Davis, B.M. and Waldor, M.K. (2015) A Genome-Wide Screen Reveals that the *Vibrio cholerae* Phosphoenolpyruvate Phosphotransferase System Modulates Virulence Gene Expression. *Infect Immun*, **83**, 3381-3395.
8. Liu, Z., Miyashiro, T., Tsou, A., Hsiao, A., Goulian, M. and Zhu, J. (2008) Mucosal penetration primes *Vibrio cholerae* for host colonization by repressing quorum sensing. *Proc Natl Acad Sci U S A*, **105**, 9769-9774.
9. Klose, K.E. and Mekalanos, J.J. (1998) Differential regulation of multiple flagellins in *Vibrio cholerae*. *J Bacteriol*, **180**, 303-316.
10. Terashima, H., Fukuoka, H., Yakushi, T., Kojima, S. and Homma, M. (2006) The *Vibrio* motor proteins, MotX and MotY, are associated with the basal body of Na-driven flagella and required for stator formation. *Mol Microbiol*, **62**, 1170-1180.
11. Yildiz, F.H. and Schoolnik, G.K. (1998) Role of rpoS in stress survival and virulence of *Vibrio cholerae*. *J Bacteriol*, **180**, 773-784.
12. Lo Scrudato, M. and Blokesch, M. (2012) The regulatory network of natural competence and transformation of *Vibrio cholerae*. *PLoS Genet*, **8**, e1002778.
13. Marvig, R.L. and Blokesch, M. (2010) Natural transformation of *Vibrio cholerae* as a tool--optimizing the procedure. *BMC Microbiol*, **10**, 155.
14. Lo Scrudato, M. and Blokesch, M. (2013) A transcriptional regulator linking quorum sensing and chitin induction to render *Vibrio cholerae* naturally transformable. *Nucleic Acids Res*, **41**, 3644-3658.
15. Lo Scrudato, M., Borgeaud, S. and Blokesch, M. (2014) Regulatory elements involved in the expression of competence genes in naturally transformable *Vibrio cholerae*. *BMC Microbiol*, **14**, 327.
16. Metzger, L.C., Stutzmann, S., Scrignari, T., Van der Henst, C., Matthey, N. and Blokesch, M. (2016) Independent Regulation of Type VI Secretion in *Vibrio cholerae* by TfoX and TfoY. *Cell Rep*, **15**, 951-958.

17. Seitz, P. and Blokesch, M. (2013) DNA-uptake machinery of naturally competent *Vibrio cholerae*. *Proc Natl Acad Sci U S A*, **110**, 17987-17992.
18. Suckow, G., Seitz, P. and Blokesch, M. (2011) Quorum sensing contributes to natural transformation of *Vibrio cholerae* in a species-specific manner. *J Bacteriol*, **193**, 4914-4924.
19. Meibom, K.L., Blokesch, M., Dolganov, N.A., Wu, C.Y. and Schoolnik, G.K. (2005) Chitin induces natural competence in *Vibrio cholerae*. *Science*, **310**, 1824-1827.
20. Borgeaud, S., Metzger, L.C., Scrignari, T. and Blokesch, M. (2015) The type VI secretion system of *Vibrio cholerae* fosters horizontal gene transfer. *Science*, **347**, 63-67.
21. Simon, R., Priefer, U. and Pühler, A. (1983) A Broad Host Range Mobilization System for In Vivo Genetic Engineering: Transposon Mutagenesis in Gram Negative Bacteria. *Bio/Technology*, **1**.
22. Ferrieres, L., Hemery, G., Nham, T., Guerout, A.M., Mazel, D., Beloin, C. and Ghigo, J.M. (2010) Silent mischief: bacteriophage Mu insertions contaminate products of *Escherichia coli* random mutagenesis performed using suicidal transposon delivery plasmids mobilized by broad-host-range RP4 conjugative machinery. *J Bacteriol*, **192**, 6418-6427.
23. De Souza Silva, O. and Blokesch, M. (2010) Genetic manipulation of Vibrio cholerae by combining natural transformation with FLP recombination. *Plasmid*, **64**, 186-195.
24. Van der Henst, C., Clerc, S., Stutzmann, S., Stoudmann, C., Scrignari, T., Maclachlan, C., Knott, G. and Blokesch, M. (2017) Molecular insights into *Vibrio cholerae*'s intra-amoebal host-pathogen interactions. *bioRxiv*.
25. Bao, Y., Lies, D.P., Fu, H. and Roberts, G.P. (1991) An improved Tn7-based system for the single-copy insertion of cloned genes into chromosomes of gram-negative bacteria. *Gene*, **109**, 167-168.
26. Nielsen, A.T., Dolganov, N.A., Otto, G., Miller, M.C., Wu, C.Y. and Schoolnik, G.K. (2006) RpoS controls the *Vibrio cholerae* mucosal escape response. *PLoS Pathog*, **2**, e109.
27. Heidelberg, J.F., Eisen, J.A., Nelson, W.C., Clayton, R.A., Gwinn, M.L., Dodson, R.J., Haft, D.H., Hickey, E.K., Peterson, J.D., Umayam, L. *et al.* (2000) DNA sequence of both chromosomes of the cholera pathogen *Vibrio cholerae*. *Nature*, **406**, 477-483.
28. Blokesch, M. (2012) Chitin colonization, chitin degradation and chitin-induced natural competence of *Vibrio cholerae* are subject to catabolite repression. *Environ Microbiol*, **14**, 1898-1912.
29. Antonova, E.S., Bernardy, E.E. and Hammer, B.K. (2012) Natural competence in *Vibrio cholerae* is controlled by a nucleoside scavenging response that requires CytR-dependent anti-activation. *Mol Microbiol*, **86**, 1215-1231.

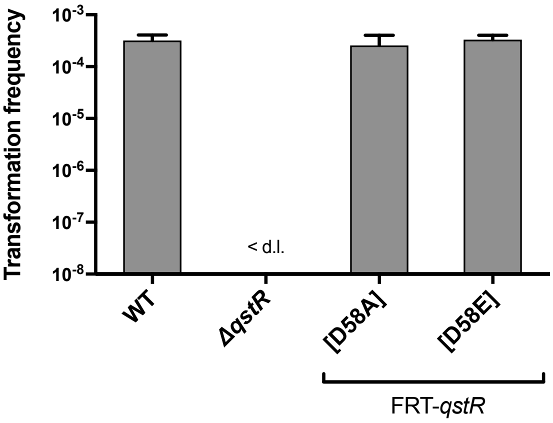

Figure S1. Mutations in the putative phosphorylation site of QstR do not affect its functionality. Transformation frequencies of strains with wild-type QstR and two QstR variants with substitutions in the putative phosphorylation site encoded in the native locus of the chromosome. All strains carry an inducible copy of *tfoX* (Tn*tfoX*) and were grown with 0.02% arabinose. Strains marked as FRT-*qstR* contain an FRT scar upstream of the *qstR* gene that does not affect transformation (see Fig. 1). The values are averages of three independent experiments with error bars representing the SD. < d.l., below detection limit.

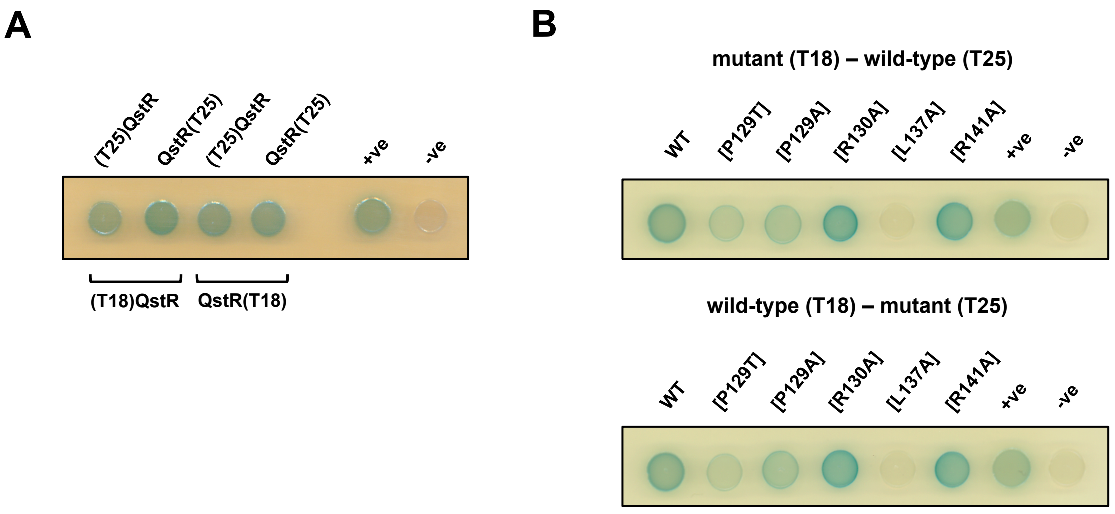

Figure S2. QstR-QstR interactions in the bacterial two-hybrid system (BACTH). (A) QstR self-interaction. Wild-type QstR was fused to either N- or C-terminus of T18 and T25 adenylate cyclase fragments and QstR interactions were examined in all four combinations. **(B)** Interactions between wild-type QstR and the indicated QstR variant. For panels A and B 5 µl of overnight culture carrying the BACTH plasmids was spotted on plates containing appropriate antibiotics, IPTG and X-gal. Plates were scanned after 24 h incubation at 30°C. The strains carrying pKT25-zip/pUT18C-zip and pUT18/pKNT25qstR served as positive (+ve) and negative (-ve) controls, respectively.

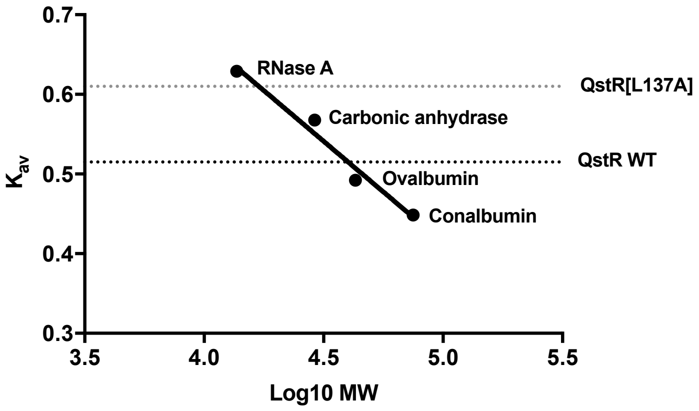

Figure S3. Purified QstR and QstR[L137A] molecular weight determination by analytical size exclusion chromatography (SEC). The standard curve was obtained by applying a set of known protein standards to a Superdex 200 10/300 GL gel filtration column (GE Healthcare) and plotting the partition coefficients (K_{av}) against the Log10 of their molecular weight (MW). The apparent molecular weight of QstR WT was 40.2 kDa and of QstR[L137A] was 17.3 kDa (theoretical values: 24.7 kDa for monomer, 49.4 kDa for dimer).

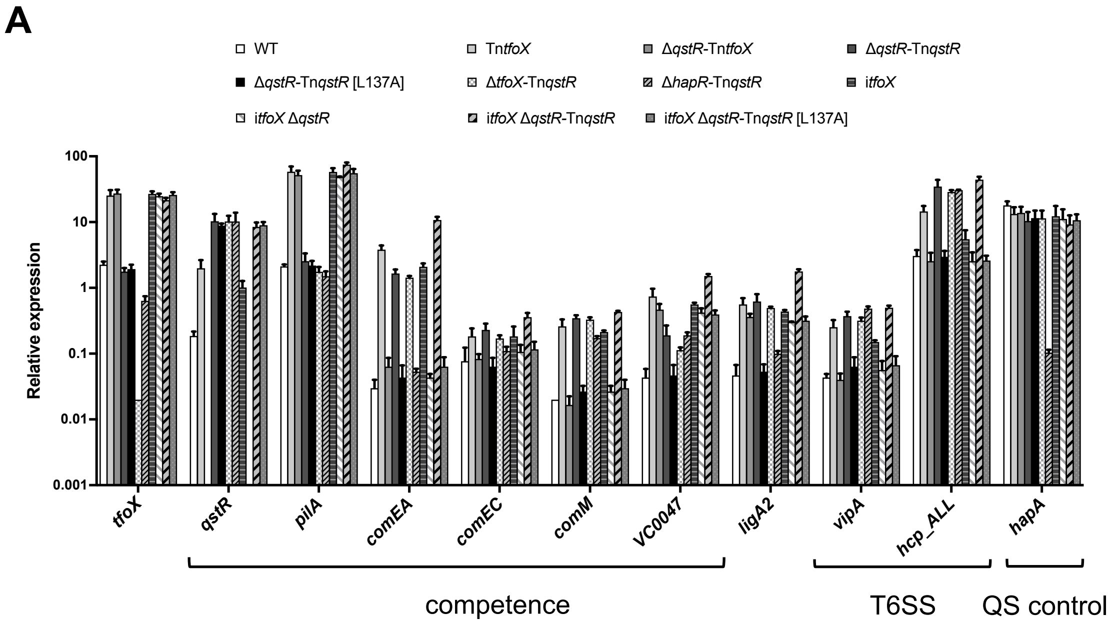

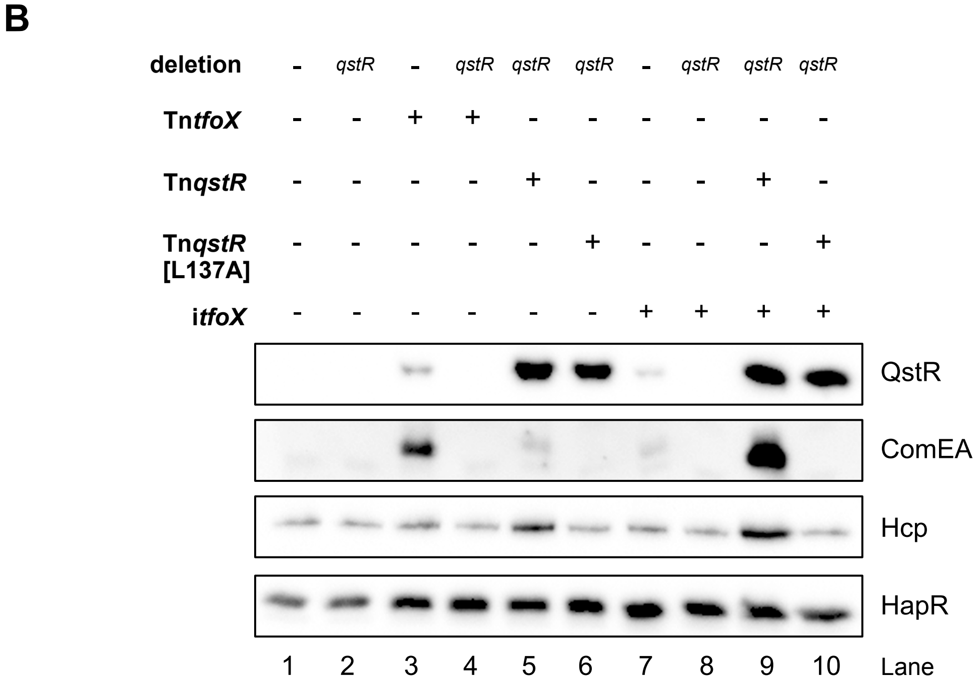

Figure S4. QstR is sufficient to induce expression of T6SS genes and a subset of competence genes. (A) The graph demonstrates expression of two representative T6SS genes (*vipA* and *hcp*) and several competence genes when TfoX, QstR or both regulators are produced. To allow co-expression of *qstR* and *tfoX*, an arabinose-inducible copy of *tfoX* at a neutral chromosomal locus (i*tfoX*) was used. Expression of *hapA* is shown as a proxy for HapR activity. The expression levels were determined by RT-qPCR and are shown as ratio to *gyrA*. The values are averages of three independent experiments with error bars representing the SD. **(B)** Protein levels of QstR, ComEA, Hcp and HapR when TfoX, QstR and TfoX together with QstR are produced. For both panels expression of t*foX* and *qstR* from the transposon (Tn) and *tfoX* from i*tfoX* was induced by 0.02% arabinose.

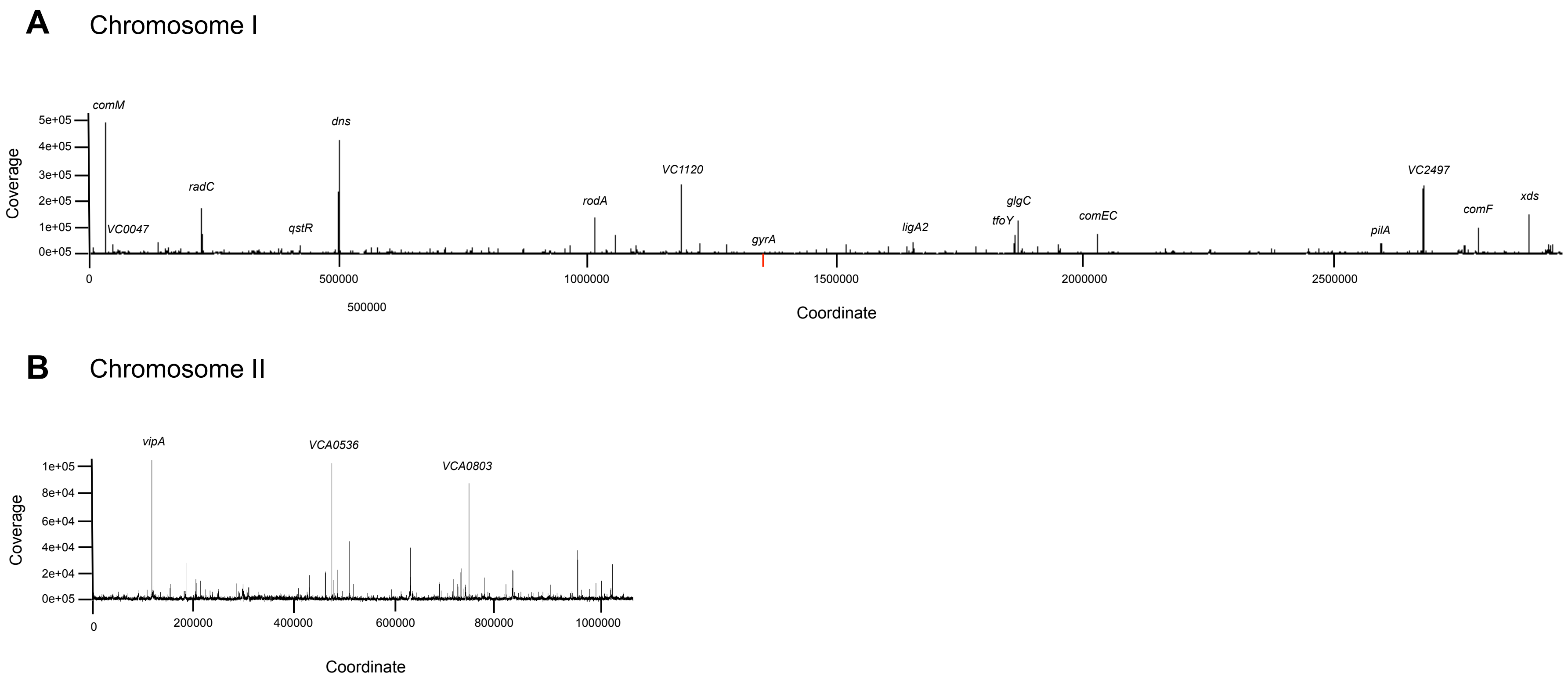

Figure S5. ChIP-seq coverage. Sequencing coverage of DNA reads obtained by chromatin immunoprecipitation with anti-QstR antibody of A1552 Tn*tfoX* lysate. The reads were mapped to the reference sequence of *V. cholerae* O1 El Tor strain N16961 chromosome I (accession number NC_002505 (27), panel **A**) and chromosome II (accession number NC_002506, panel **B**). The annotated peaks were validated by ChIP-qPCR. The approximate position of *gyrA* (*VC1258*), which was used in ChIP-qPCR experiment as background control, is indicated by the red line.

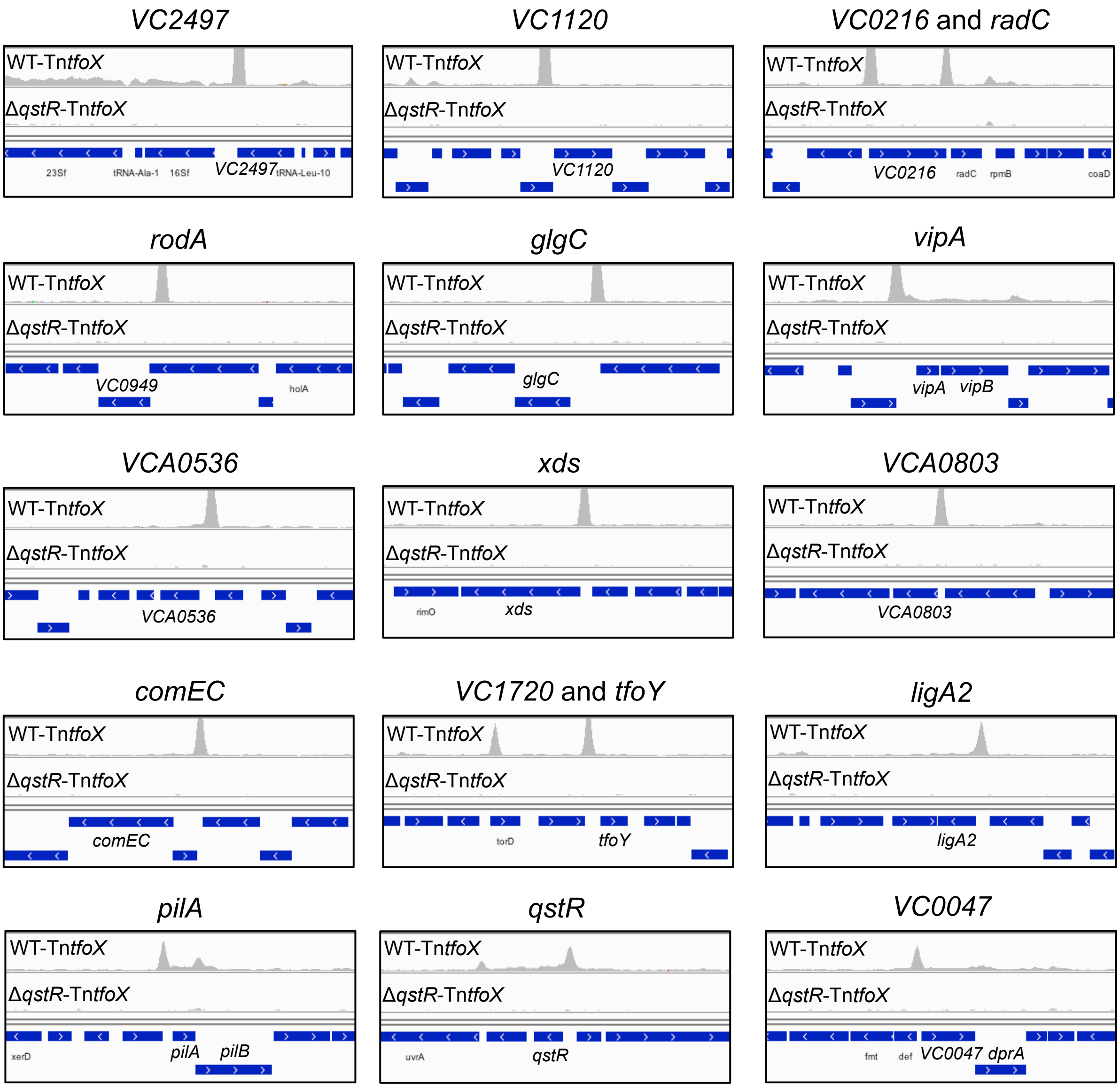

Figure S6. ChIP-seq peaks. Zoomed in coverage of DNA reads for DNA obtained by chromatin immunoprecipitation with anti-QstR antibody coupled with sequencing (ChIP-seq) for A1552-Tn*tfoX* (WT) and A1552Δ*qstR*-Tn*tfoX* strains. The reads were mapped to the reference sequence of *V. cholerae* O1 El Tor strain N16961 (accession number NC_002505 and NC_002506 for chromosome I and II, respectively (27)). The panels show QstR dependent enrichment of DNA fragments for the 15 top peaks identified by ChIP-seq analysis (peaks for *comM*, *dns* and *comF* shown in Fig. 6) and the peaks upstream of *ligA2*, *pilA*, *qstR,* and *VC0047*. The scale of the y-axis is set to the same level for all panels (0-50,000).

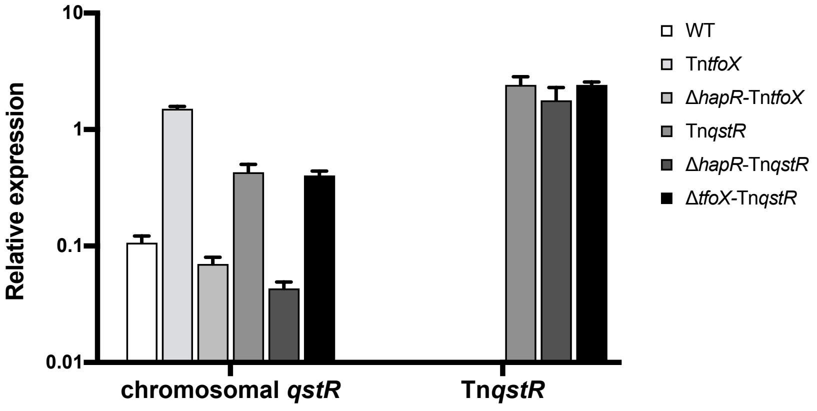

Figure S7. QstR is auto-regulated in a HapR-dependent manner. The graph shows expression of chromosomal *qstR* (primers annealing downstream of the native *qstR* gene) and *qstR* produced from the transposon (primers specific for amplification of the 5' end of the mRNA of *qstR* expressed from the P_{BAD} promoter) relative to *gyrA.* The data were obtained by RT-qPCR of strain A1552 (WT) and derivatives carrying either Tn*tfoX* or Tn*qstR*. All strains were grown with 0.02% arabinose to induce either *tfoX* or *qstR*. The values are averages of three independent experiments with error bars representing the SD.

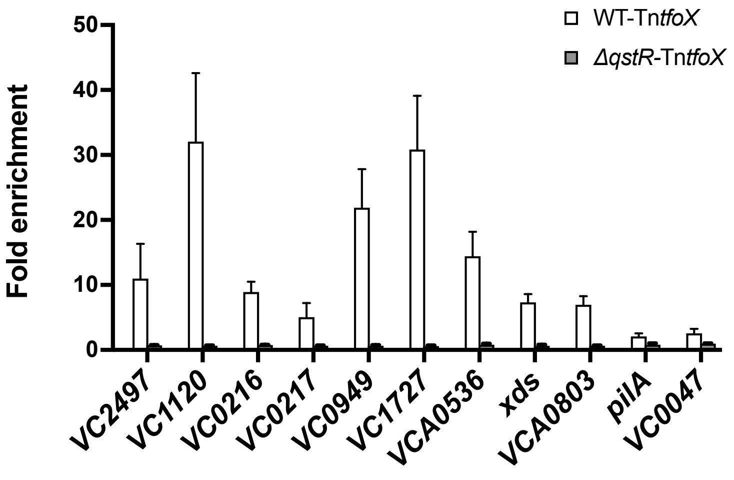

Figure S8. ChIP-seq peaks validation by qPCR. QstR-dependent enrichment of DNA fragments upstream of genes expression of which did not appear QstR-dependent in the RNA-seq data (Supplementary file 1) determined by qPCR using ChIP DNA. The fold enrichment was determined by dividing 'Input %' of the target by 'Input %' of the background (fragment upstream of *gyrA)*. The values are averages of three independent experiments with error bars representing the SD.

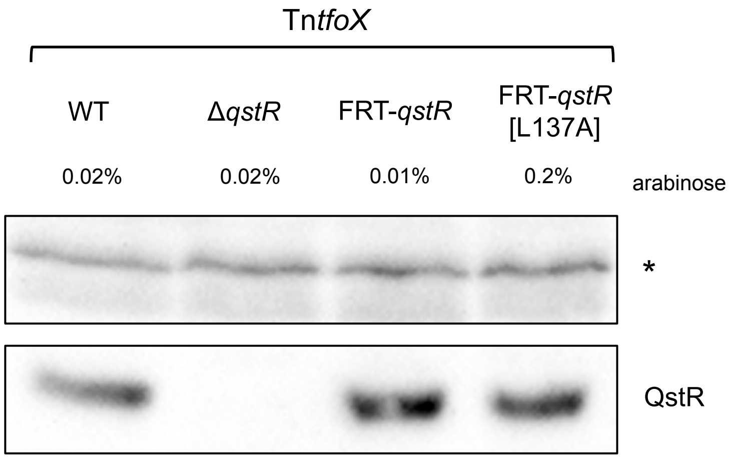

Figure S9. Similar protein levels of QstR and QstR[L137A] in lysates of FRT-*qstR* and FRT-*qstR*[L137A] strains used for ChIP-qPCR experiment. The cultures for ChIP-qPCR experiment were grown with the indicated concentration of inducer to ensure comparable levels of QstR. QstR was visualized through Western blotting analysis with anti-QstR antibodies. The higher molecular weight cross-reaction band served as a loading control (*).

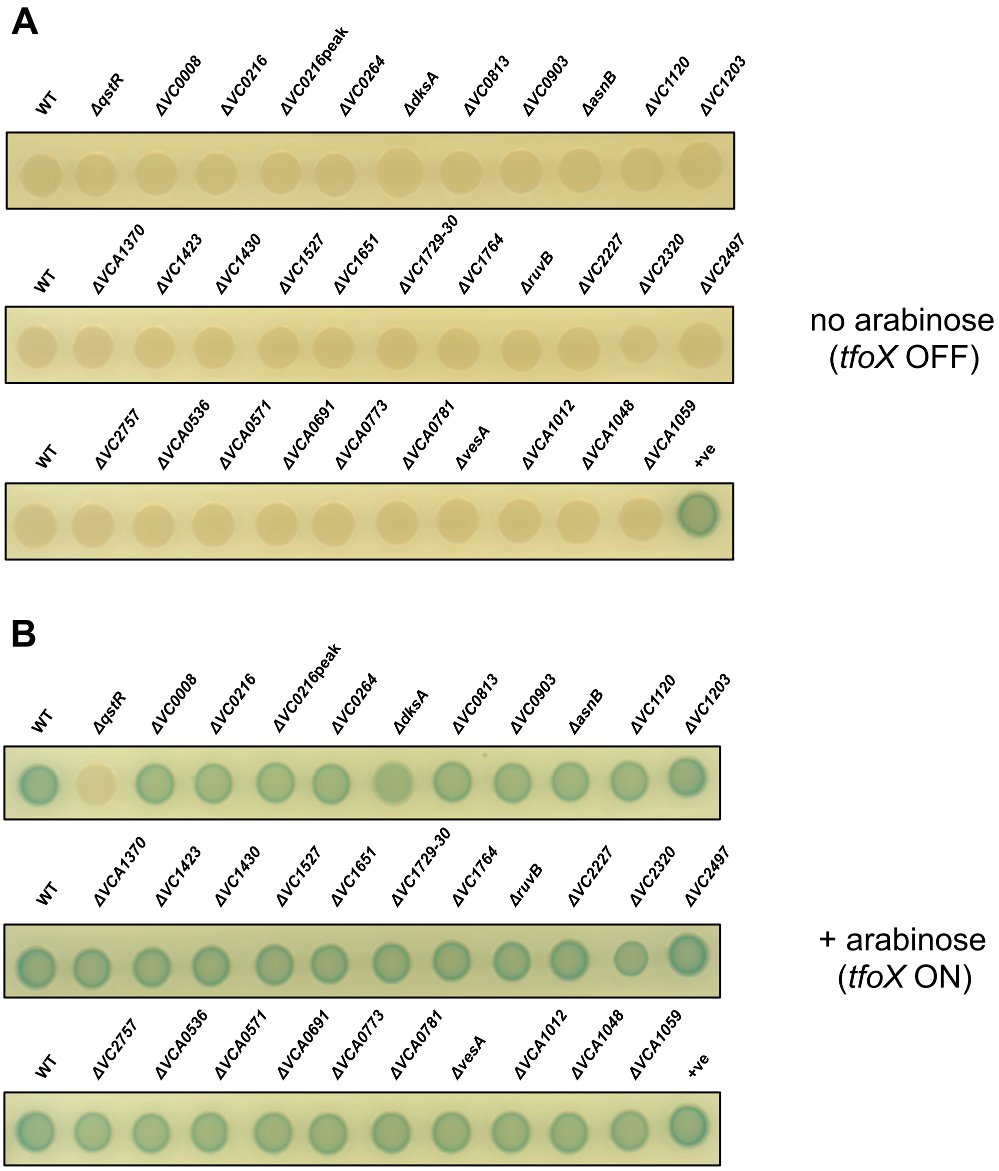

Figure S10. Deletion of genes adjacent to ChIP-seq peaks does not affect *comEA* expression. The genes potentially affected by QstR binding were deleted in the *comEA-lacZ* reporter strain (A1552*ΔlacZ-comEA-lacZ*::FRT-Tn*tfoX*). The overnight culture of each strain was diluted 10-fold and 5 µl was spotted on LB X-gal plates without **(A)** and with **(B)** 0.02% arabinose to induce *tfoX* expression. Plates were scanned after 24 h incubation at 37°C. The strain constitutively producing LacZ due to an insertion of the *mariner* transposon upstream of the *lacZ* gene was used as positive control (+ve).

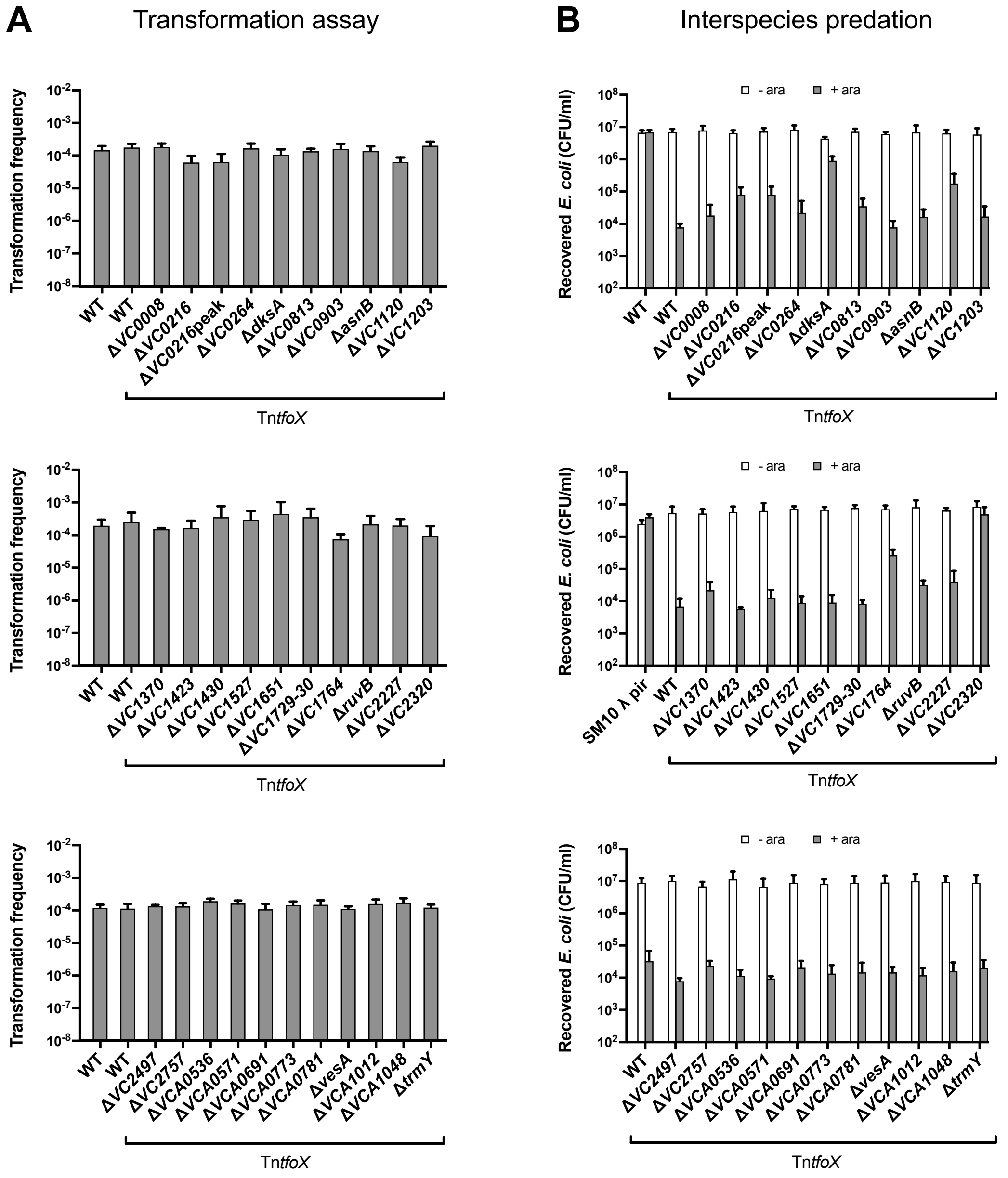

Figure S11. Deletion of genes adjacent to ChIP-seq peaks does not affect QstR dependent phenotypes. The genes potentially affected by QstR binding were deleted in the A1552-Tn*tfoX* strain and tested for transformation **(A)** and T6SS-mediated killing of an *E. coli* prey **(B)**. (A) Transformation frequencies of the mutant strains were assessed in the chitin-dependent transformation assay. (B) *V. cholerae* strains were co-cultured with *E. coli* on LB agar plates without (- ara) or with 0.2% arabinose (+ ara). The survival of the recovered prey is represented as the number of colony forming units (CFU) per milliliter. For panels A and B the values are averages of three independent experiments with error bars representing the SD.

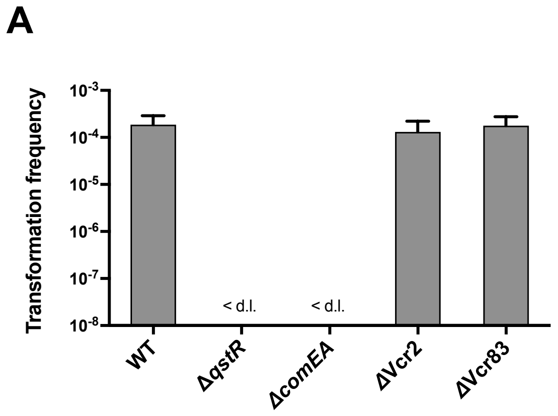

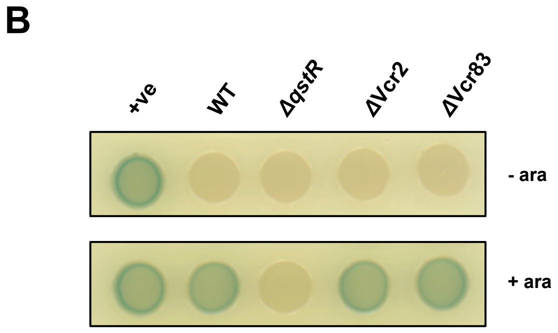

Figure S12. Deletion of two small RNAs adjacent to the QstR-dependent ChIP-seq peaks does not affect transformation or *comEA* expression. (A) Transformation frequencies of strains deleted for Vcr2 or Vcr83 assessed in a chitin-dependent transformation assay. The values are averages of three independent experiments (±SD). < d.l., below detection limit. **(B)** Strains deleted for Vcr2 or Vcr83 and carrying the *comEA-lacZ* reporter construct were spotted on LB X-gal plates without (- ara) or with 0.02% arabinose (+ ara). The plates were scanned after 24 h incubation at 37°C. The strain constitutively producing LacZ due to an insertion of the *mariner* transposon upstream of *lacZ* served as positive control (+ve).

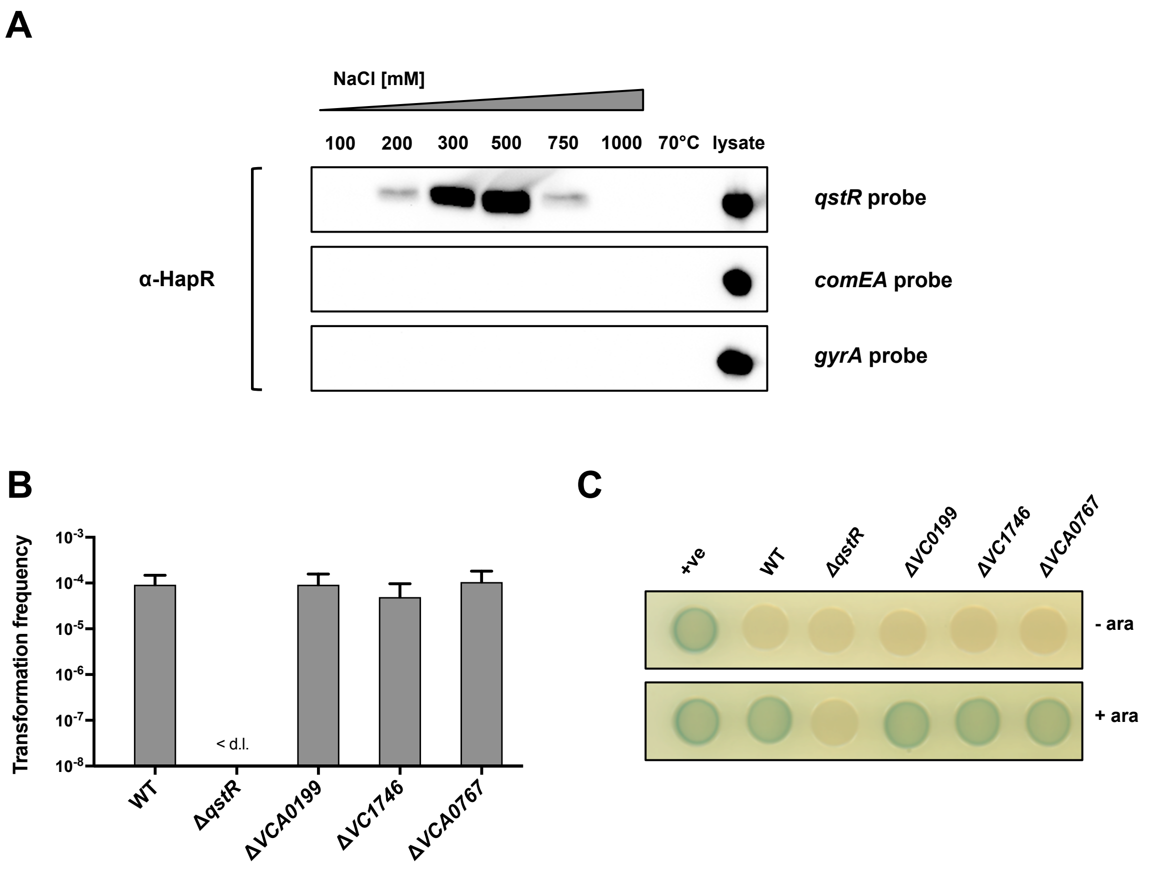

Figure S13. Deletion of genes encoding proteins identified at the *comEA* promoter in the DNA affinity pull-down assay does not affect transformation or *comEA* expression. (A) Western blot showing detectable HapR in the elution fractions of the *qstR* probe but not the *comEA* and *gyrA* probes. The diluted lysate of strain A1552-Tn*tfoX* used for incubation with the probes in the DNA affinity pull down assay was used as control. **(B)** Transformation frequencies of strains deleted for the indicated gene and assessed in a chitin-dependent transformation assay. The values are averages of three independent experiments (±SD). < d.l., below detection limit. **(C)** Strains deleted for the indicated genes and carrying the *comEA*-*lacZ* reporter construct were spotted on LB X-gal plates without (- ara) or with 0.02% arabinose (+ ara). The plates were scanned after 24 h incubation at 37°C. The strain constitutively producing LacZ due to an insertion of the *mariner* transposon upstream of *lacZ* served as positive control (+ve).

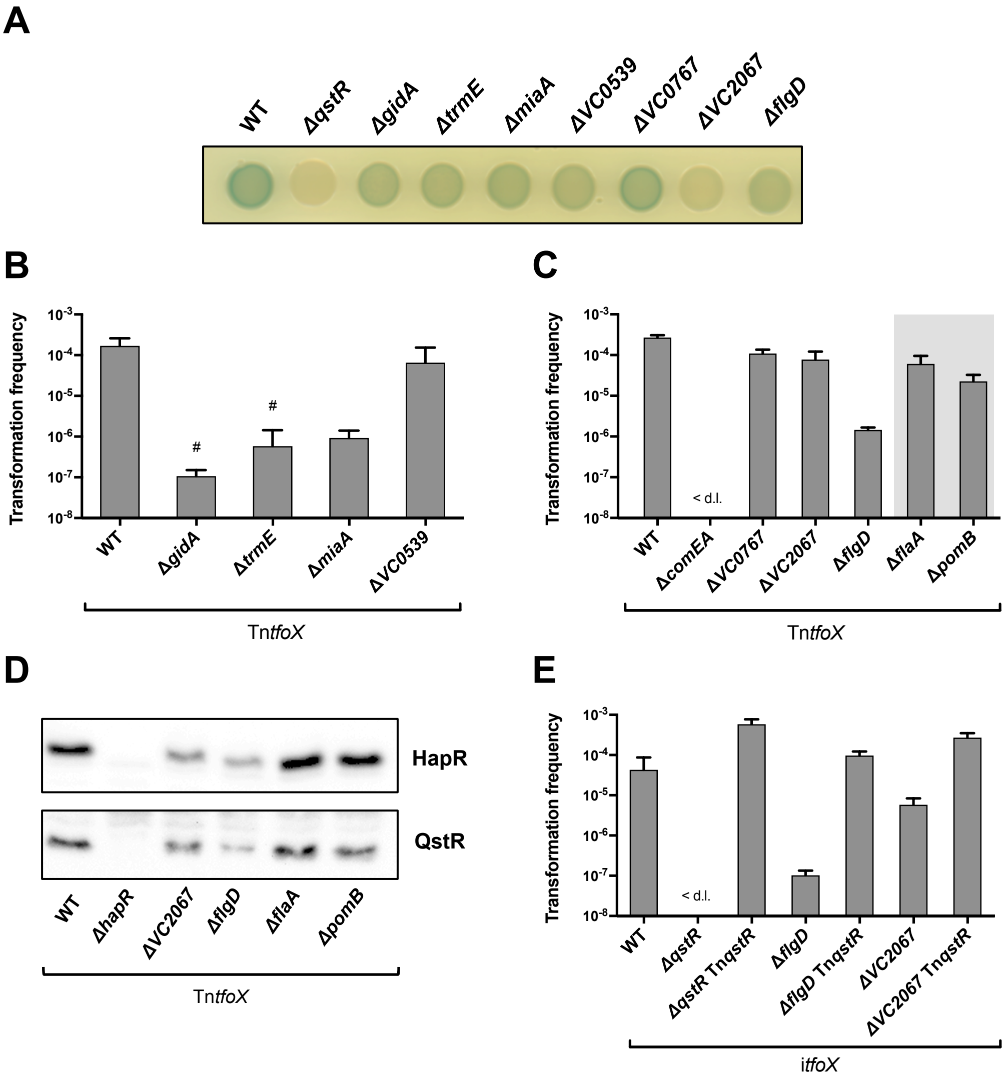

Figure S14. Effect of deletion of genes identified in the transposon mutagenesis screen on *comEA* expression and transformation. (A) Overnight cultures of strains with the indicated gene deleted in the *comEA-lacZ* reporter strain were spotted on LB X-gal plates with 0.02% arabinose. The plates were scanned after 24 h incubation at 37°C. **(B) and (C)** Transformation frequencies of strains carrying the deletion of genes identified in the first (B) and second (C) screen assessed in a chitin-independent transformation assay. All strains carried arabinose inducible *tfoX* and were grown with inducer (0.02% arabinose). Transformation of strains deleted for known motility genes (*flaA* and *pomB*) are highlighted by the shaded box. **(D)** Reduced HapR and QstR protein levels in Δ*VC2067* and Δ*flgD* strains compared to the wild-type, Δ*flaA* and Δ*pomB* strains. All strains carried Tn*tfoX* and were grown in culture medium supplemented with 0.02% arabinose. **(E)** Transformation defect of the *flgD*-minus strain is restored when inducible QstR is co-produced with TfoX. All strains carried an inducible copy of *tfoX* (i*tfoX*) and were grown with 0.02% arabinose. For panels B, C and E values shown are averages of three independent experiments with error bars representing the SD. < d.l., below the detection limit; #, transformation frequency below the detection limit in one (Δ*trmE*) or two (Δ*gidA*) out of three experiments; the detection limit was therefore used to calculate the average.
